# Supplementary material for: The protective effect of iron isomaltoside on myocardial ischemia-reperfusion injury via the suppression of KLF4/NF-κB signaling
Source: PLoS One. 2025 Jun 5;20(6):e0323247. doi: 10.1371/journal.pone.0323247 (PMC12140197; doi:10.1371/journal.pone.0323247)
Supplement: Fig S1 — IIM supplementation decreased ferritin concentration in the IR group and IR + IIM group (p < 0.05, n = 3). Representative picture of density analysis for KLF4 in rat heart tissues of each group (n = 3). Full-length blots/gels are presented in supplementary for Fig S1. (C) TTC results of myocardial infarction in rats in indicated groups. 1–3 were the IR group, 4–6 were the IR + IIM group. The myocardial slices showed clear infarct areas with infarct size attached. (D) HE staining for inflammatory infiltration of myocardial cells. (PDF) [file pone.0323247.s001.pdf]

Fig1C BAX

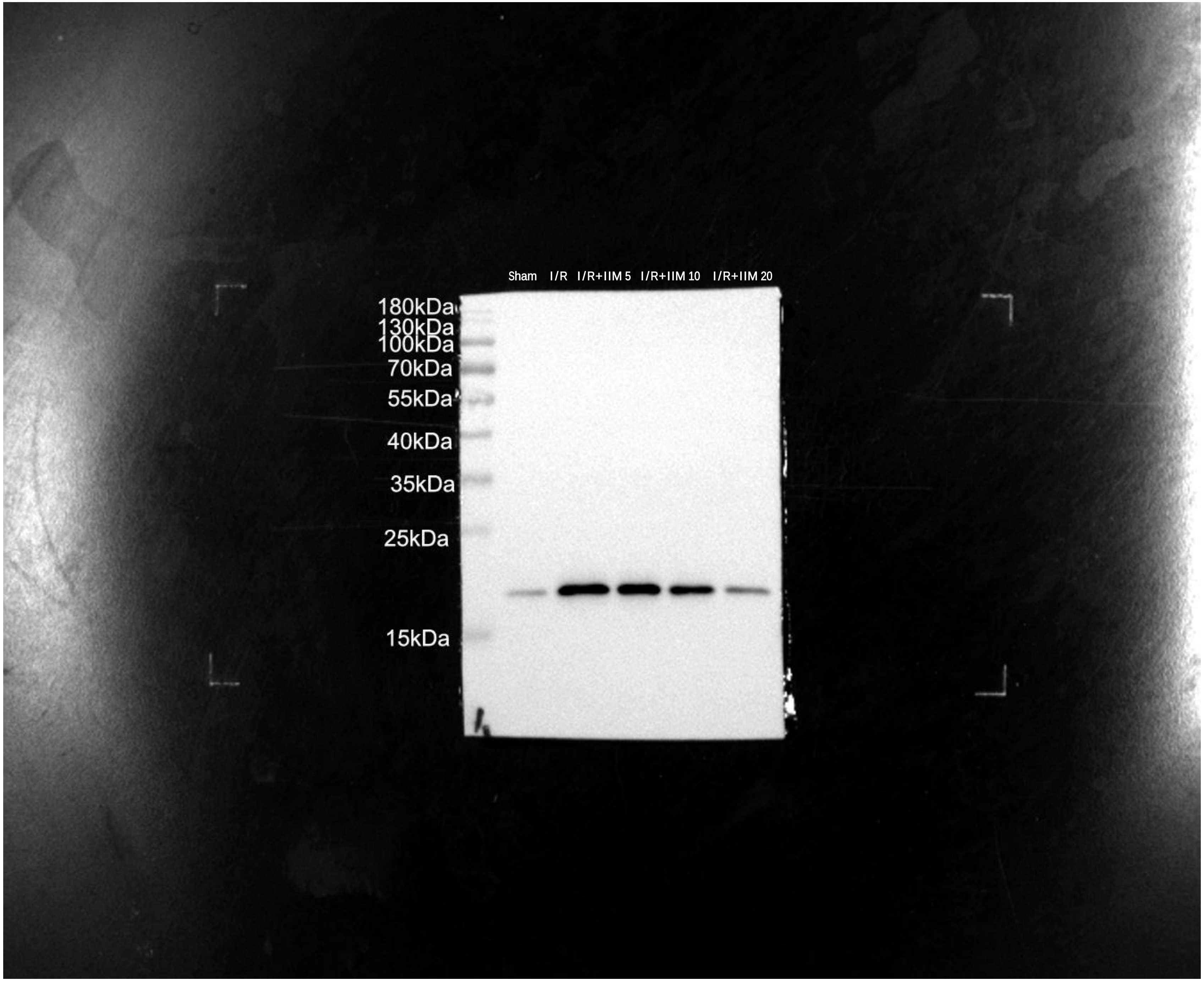
Sham
I/R
I/R+IIM 5
I/R+IIM 10
I/R+IIM 20
180kDa
130kDa
100kDa
70kDa
55kDa
40kDa
35kDa
25kDa
15kDa

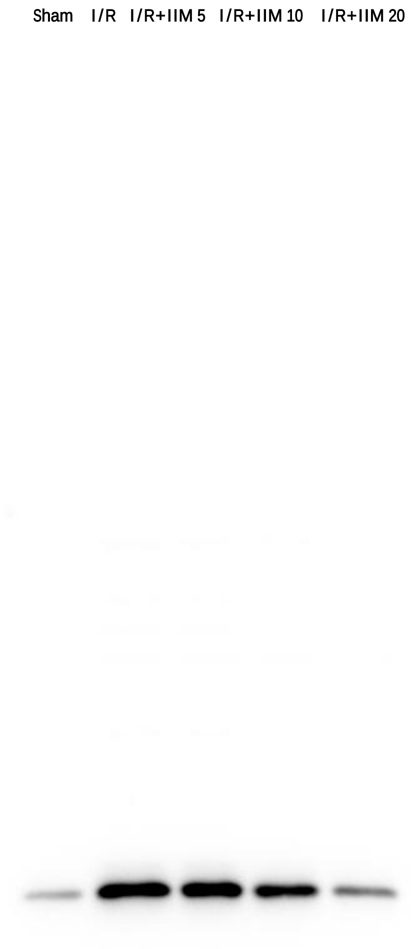
Sham
I/R
I/R+IIM 5
I/R+IIM 10
I/R+IIM 20

Fig1C-actin

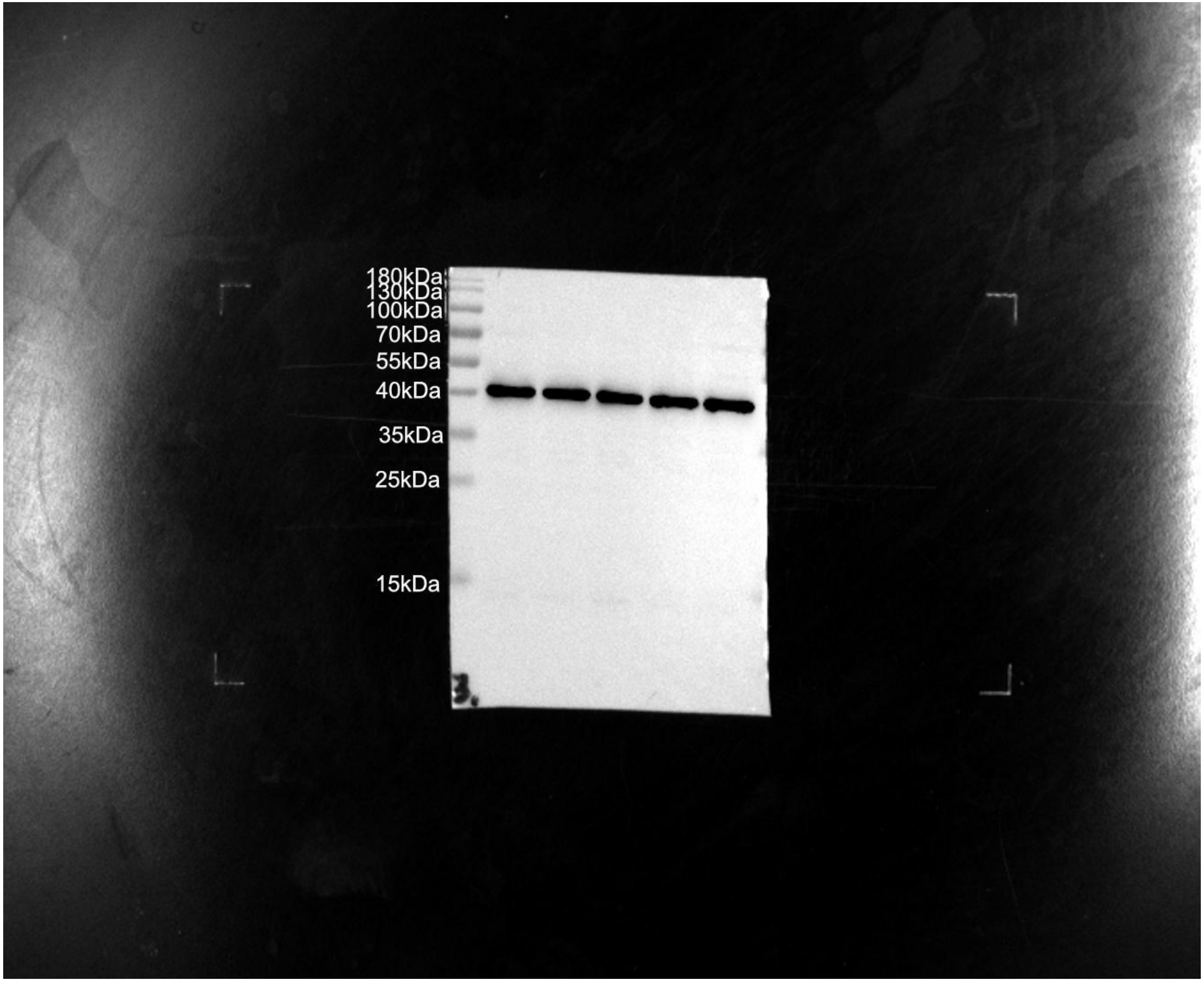

Sham I/R I/R+IIM 5 I/R+IIM 10 I/R+IIM 20

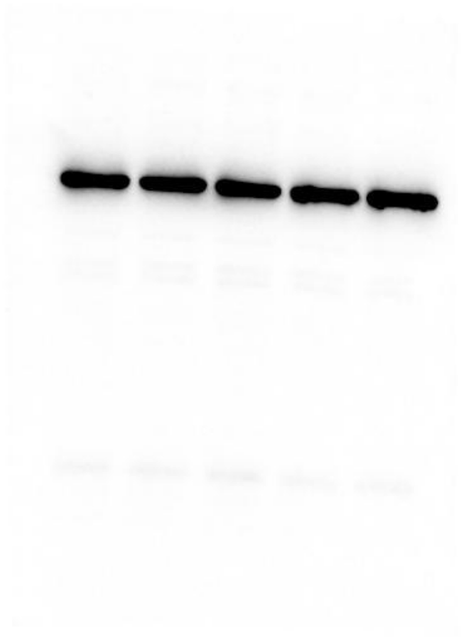

Fig1C-CAS3

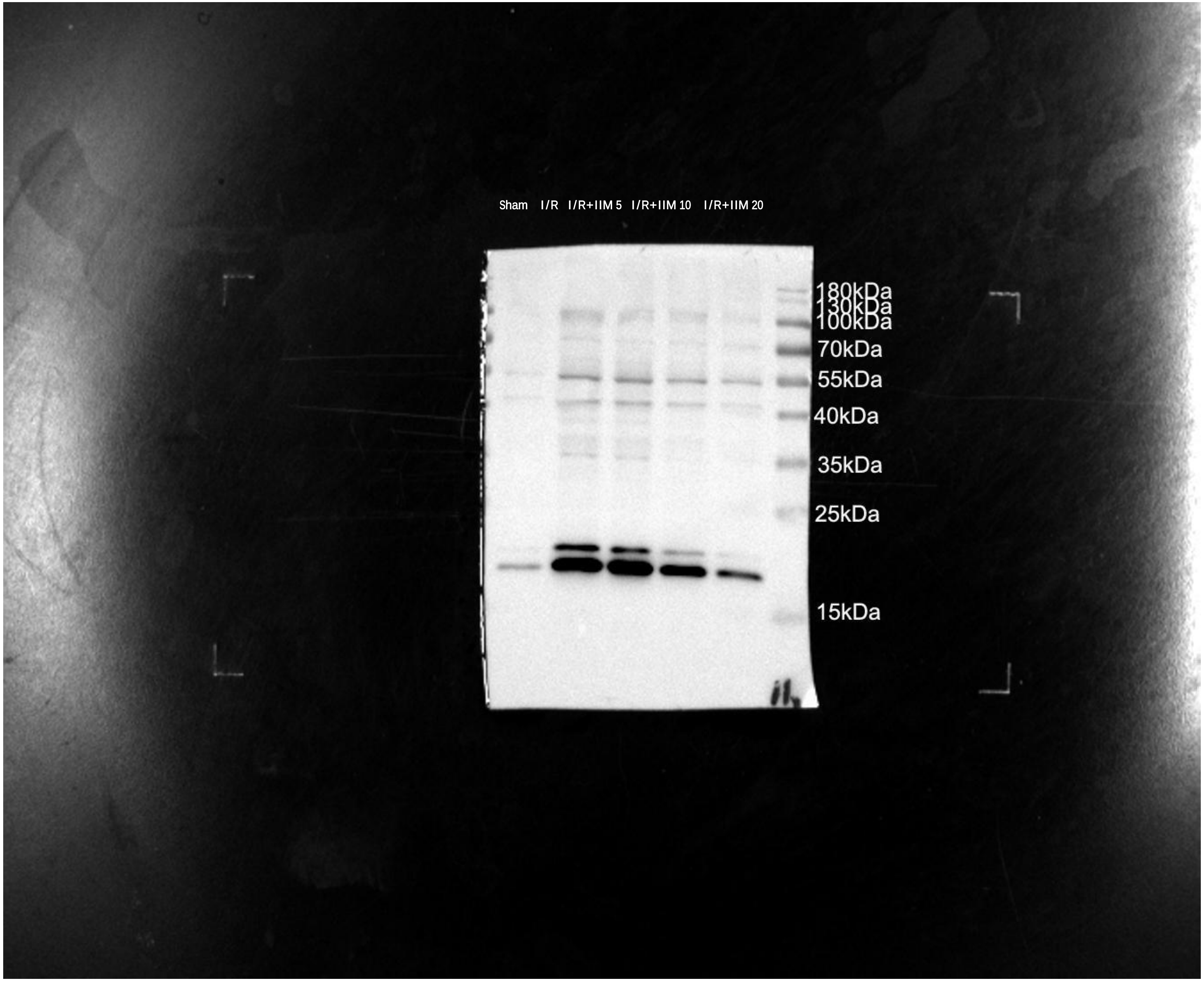
Sham
I/R
I/R+IIM 5
I/R+IIM 10
I/R+IIM 20
180kDa
130kDa
100kDa
70kDa
55kDa
40kDa
35kDa
25kDa
15kDa

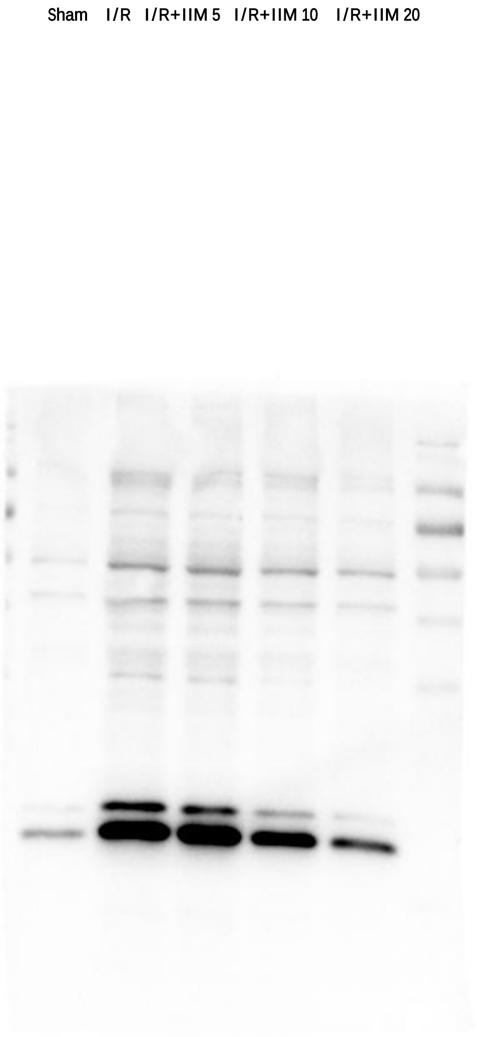
Sham
I/R
I/R+IIM 5
I/R+IIM 10
I/R+IIM 20

Fig1C-Bcl-2

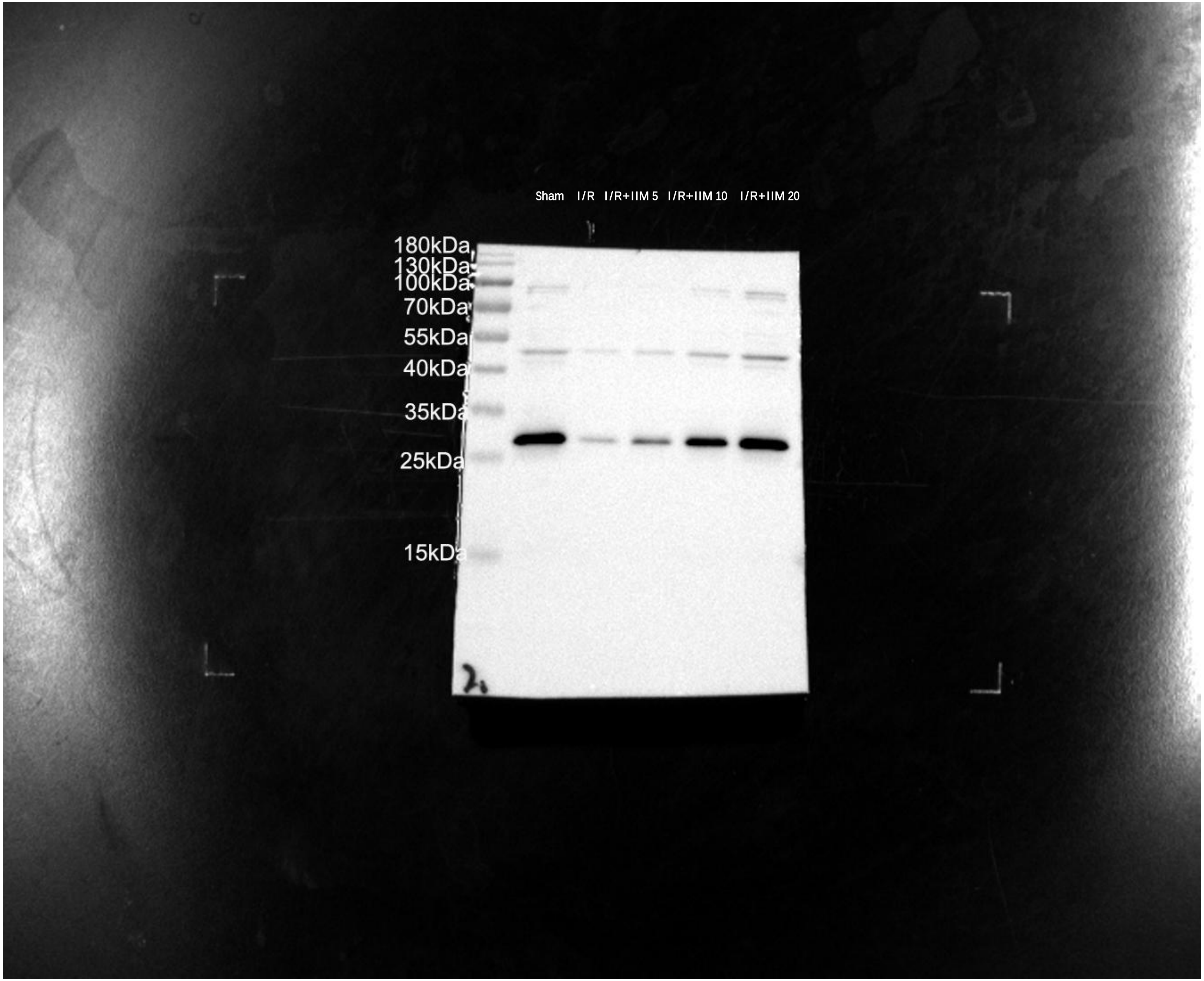

Sham
I/R
I/R+IIM 5
I/R+IIM 10
I/R+IIM 20
180kDa
130kDa
100kDa
70kDa
55kDa
40kDa
35kDa
25kDa
15kDa

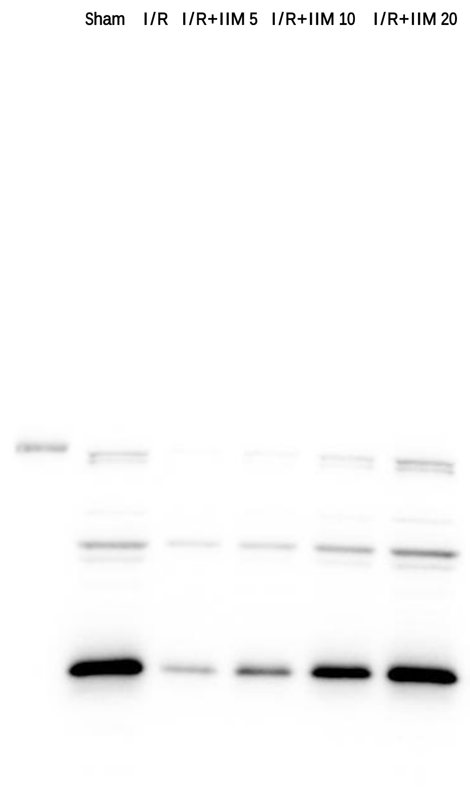
Sham
I/R
I/R+IIM 5
I/R+IIM 10
I/R+IIM 20

Fig2B IL-1b

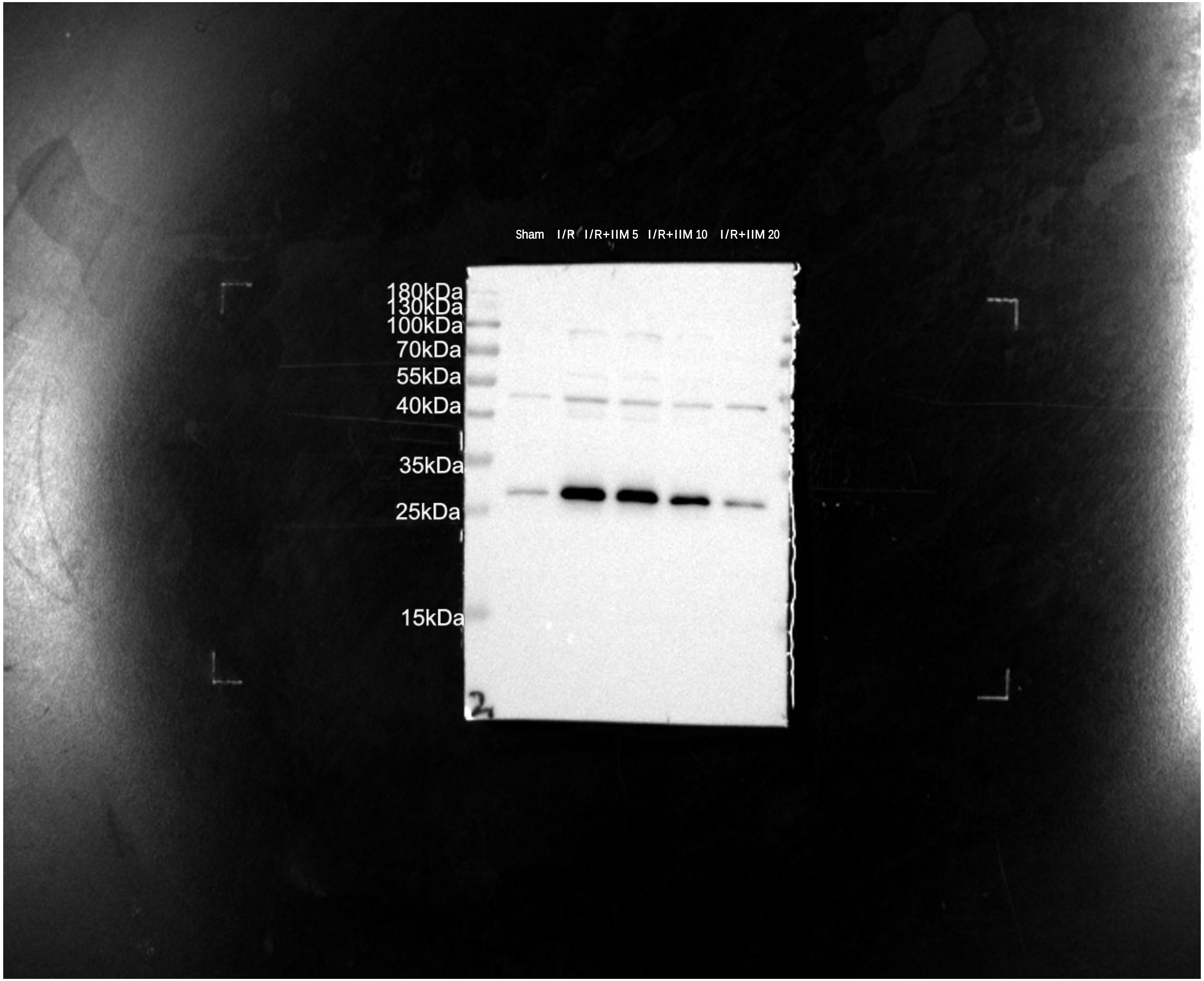
Sham
I/R
I/R+IIM 5
I/R+IIM 10
I/R+IIM 20
180kDa
130kDa
100kDa
70kDa
55kDa
40kDa
35kDa
25kDa
15kDa

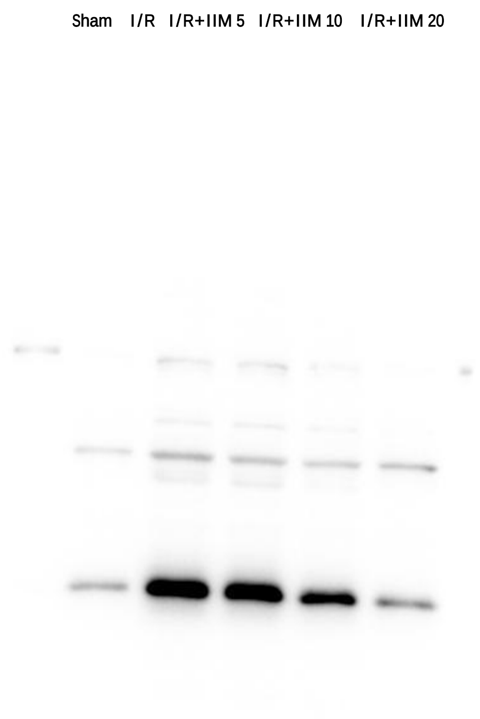
Sham
I/R
I/R+IIM 5
I/R+IIM 10
I/R+IIM 20

Fig2B IL-6

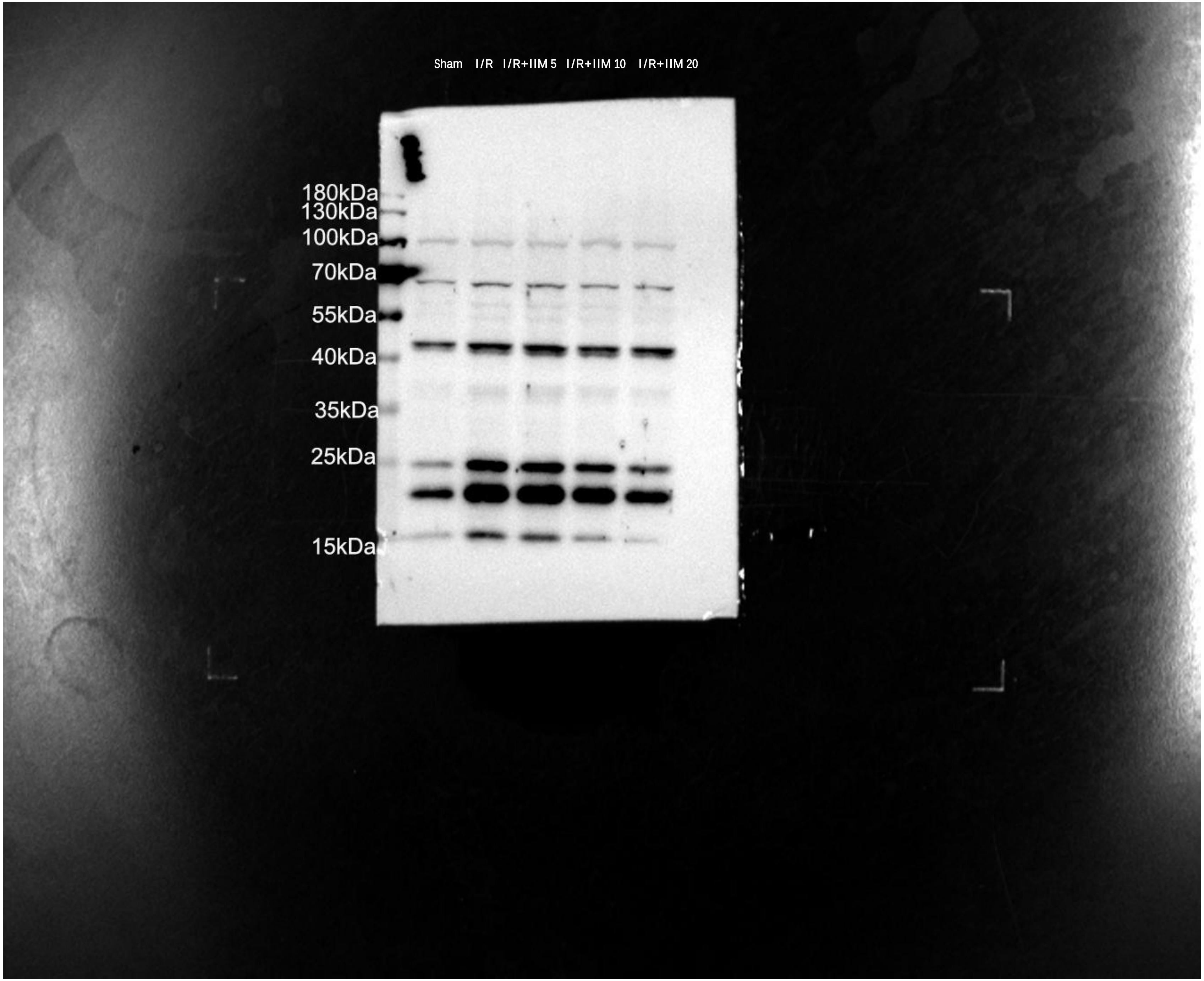
Sham
I/R
I/R+IIM 5
I/R+IIM 10
I/R+IIM 20
180kDa
130kDa
100kDa
70kDa
55kDa
40kDa
35kDa
25kDa
15kDa

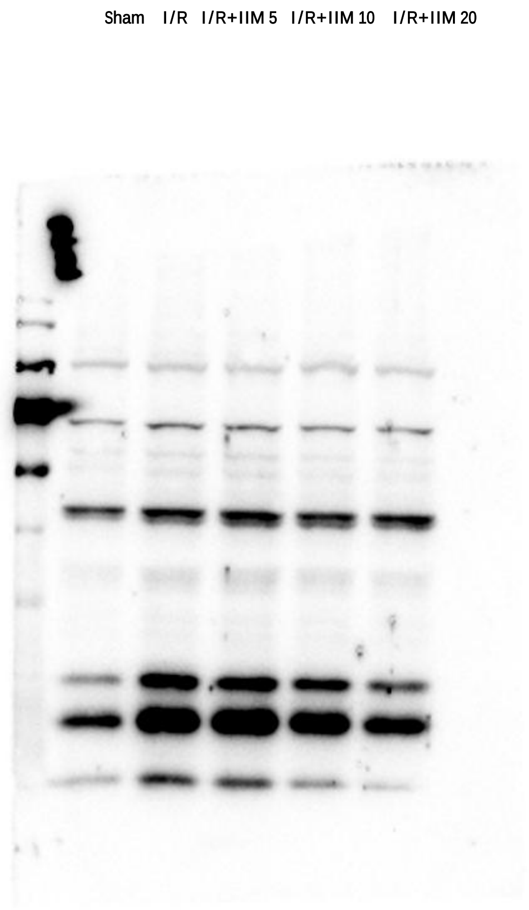
Sham
I/R
I/R+IIM 5
I/R+IIM 10
I/R+IIM 20

Fig2B ACTIN

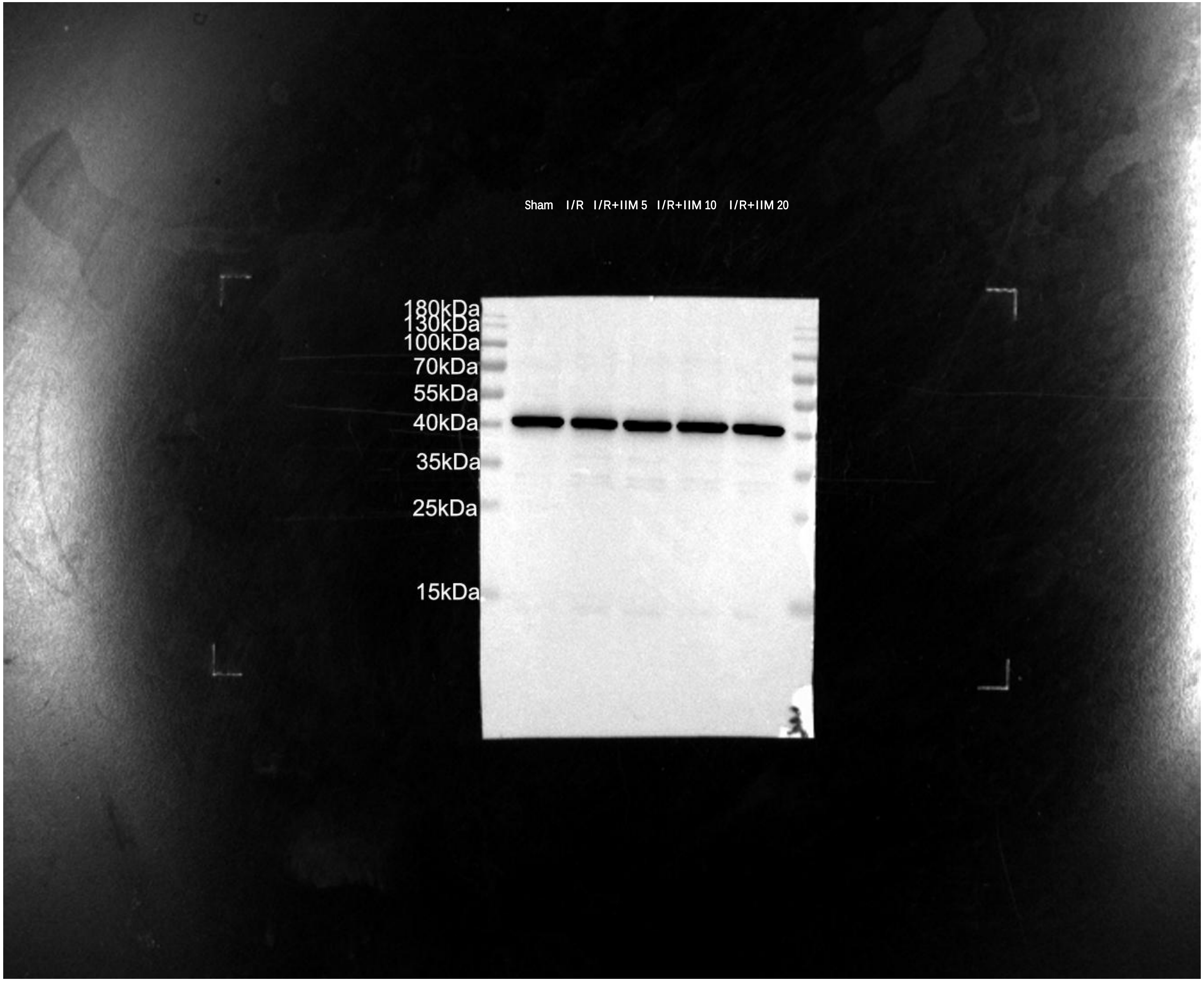

Sham
I/R
I/R+IIM 5
I/R+IIM 10
I/R+IIM 20
180kDa
130kDa
100kDa
70kDa
55kDa
40kDa
35kDa
25kDa
15kDa

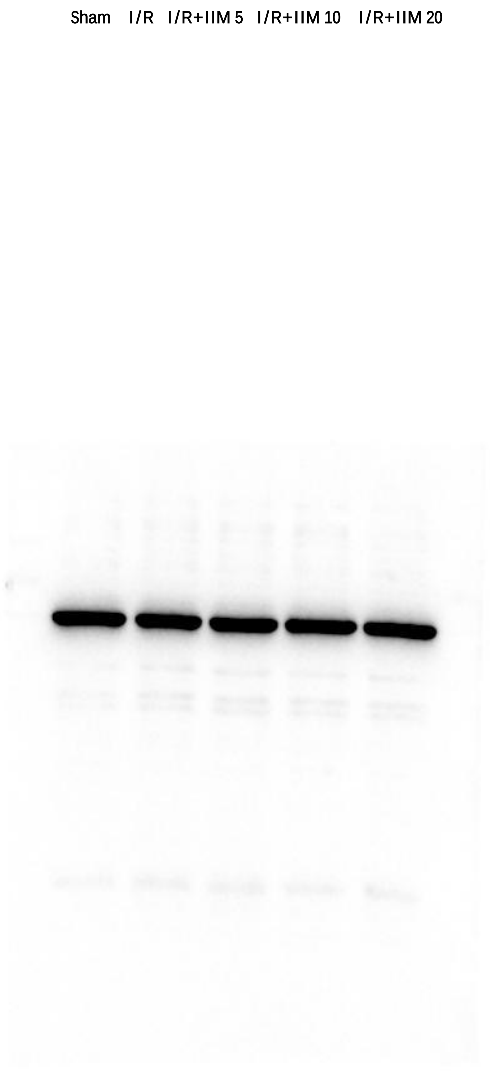
Sham
I/R
I/R+IIM 5
I/R+IIM 10
I/R+IIM 20

Fig2B TNFa

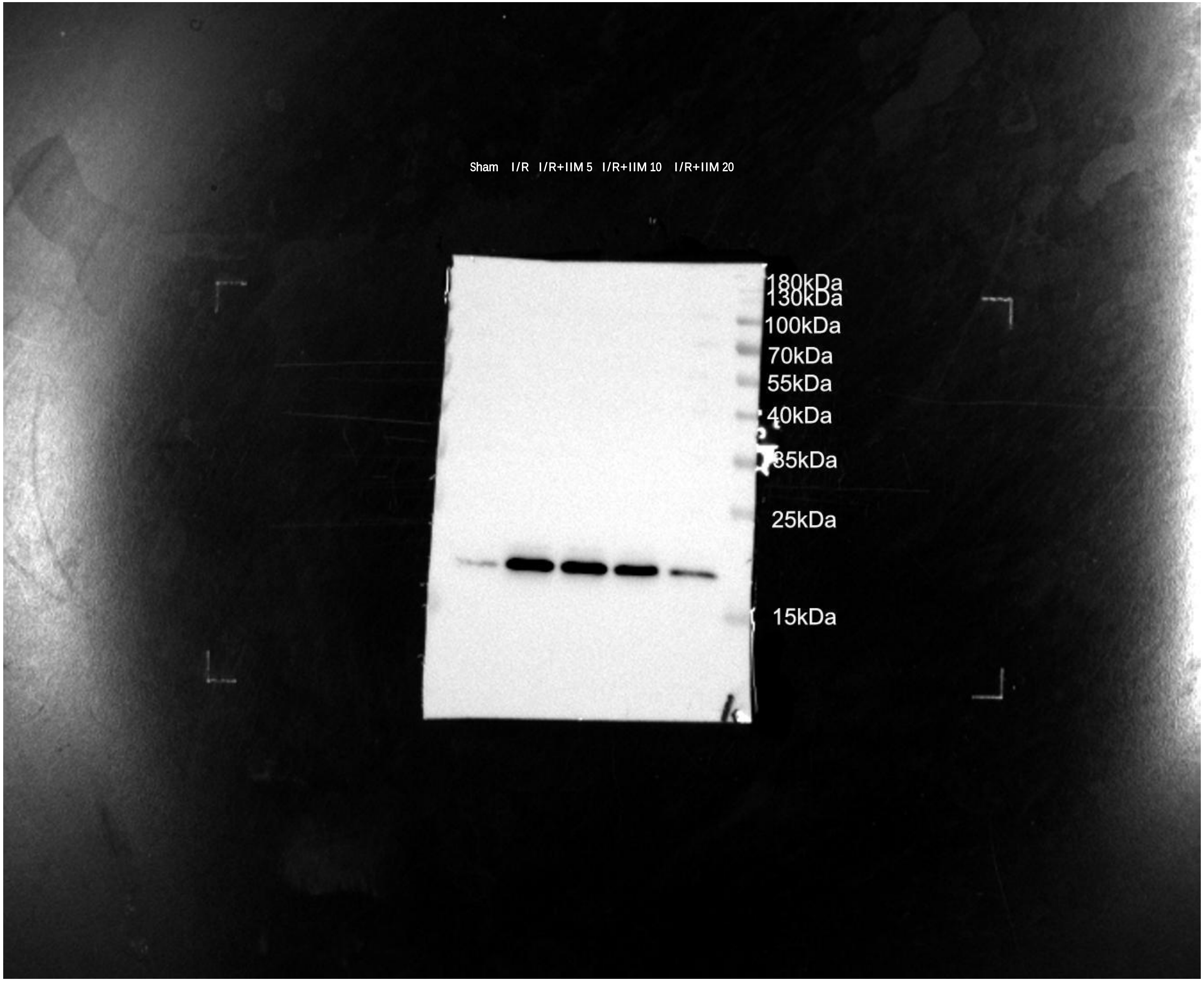
Sham I/R I/R+IIM 5 I/R+IIM 10 I/R+IIM 20
180kDa
130kDa
100kDa
70kDa
55kDa
40kDa
35kDa
25kDa
15kDa

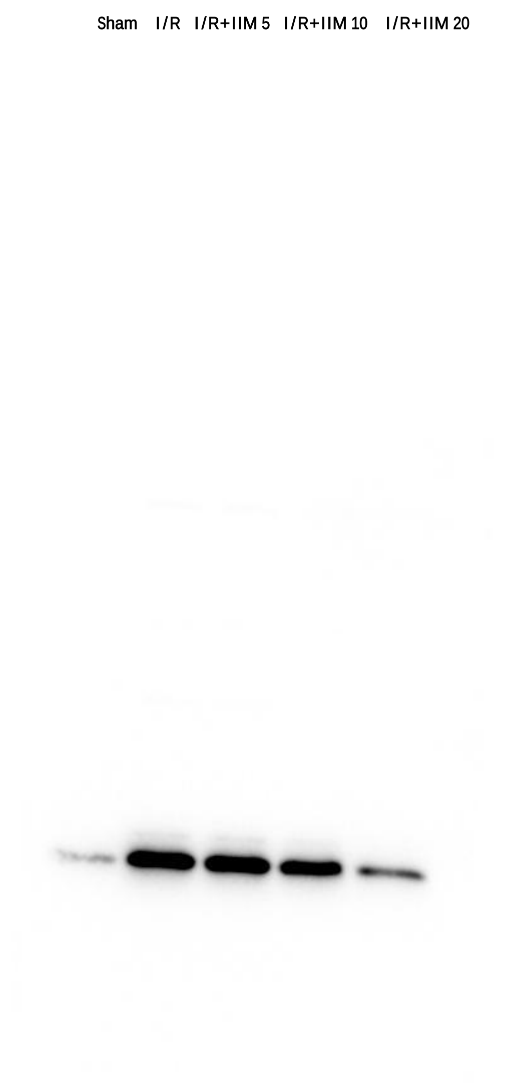
Sham
I/R
I/R+IIM 5
I/R+IIM 10
I/R+IIM 20

Fig2C Beclin1

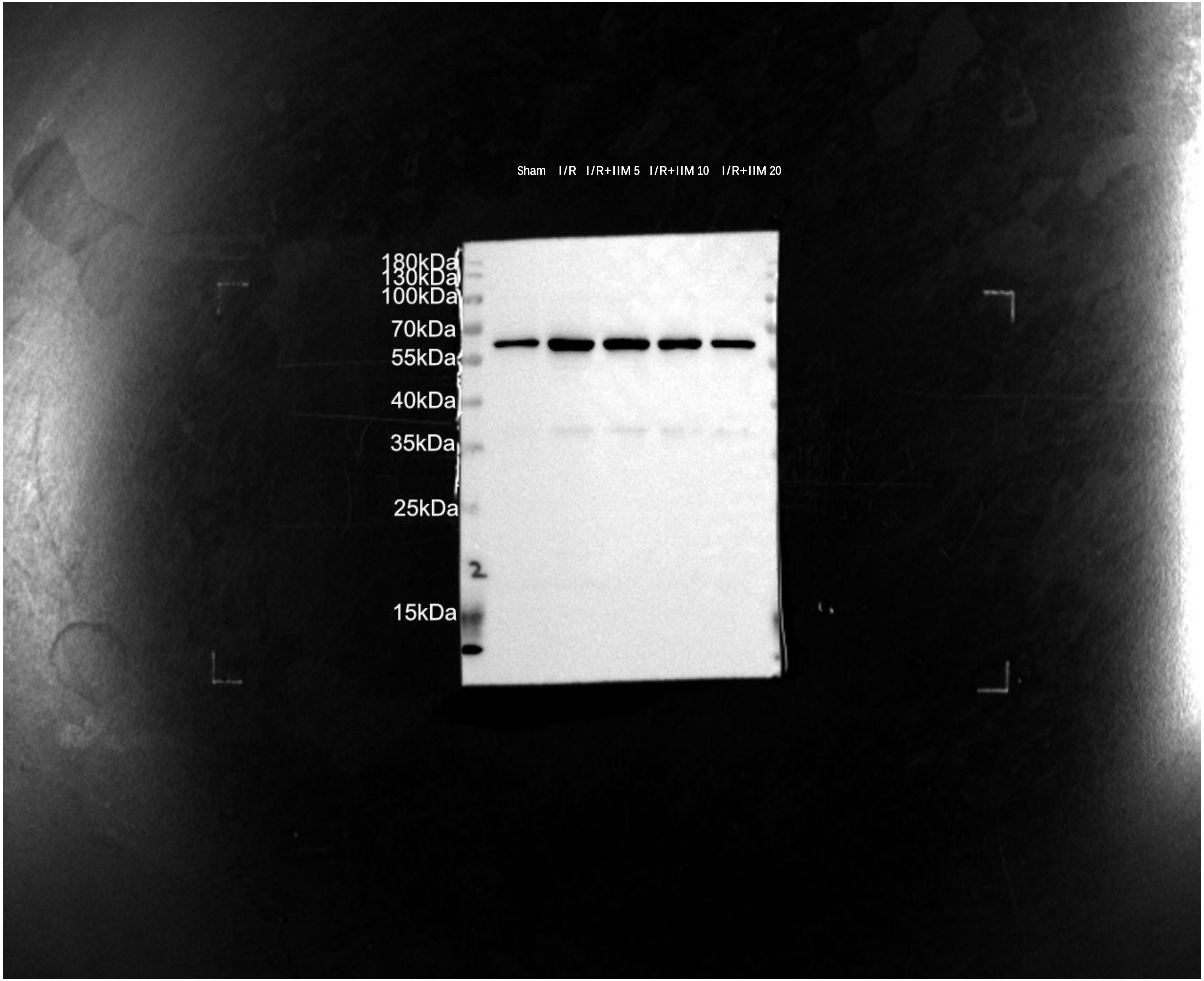

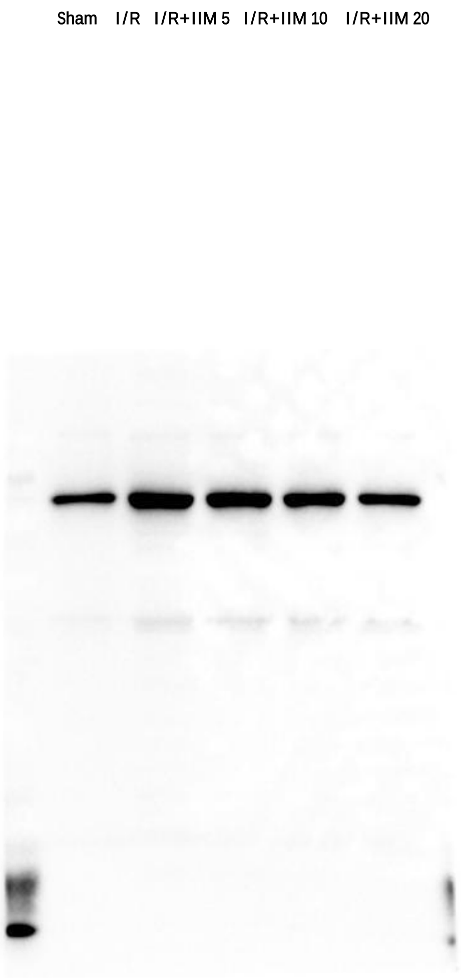
Sham
I/R
I/R+IIM 5
I/R+IIM 10
I/R+IIM 20

Fig2C LC3

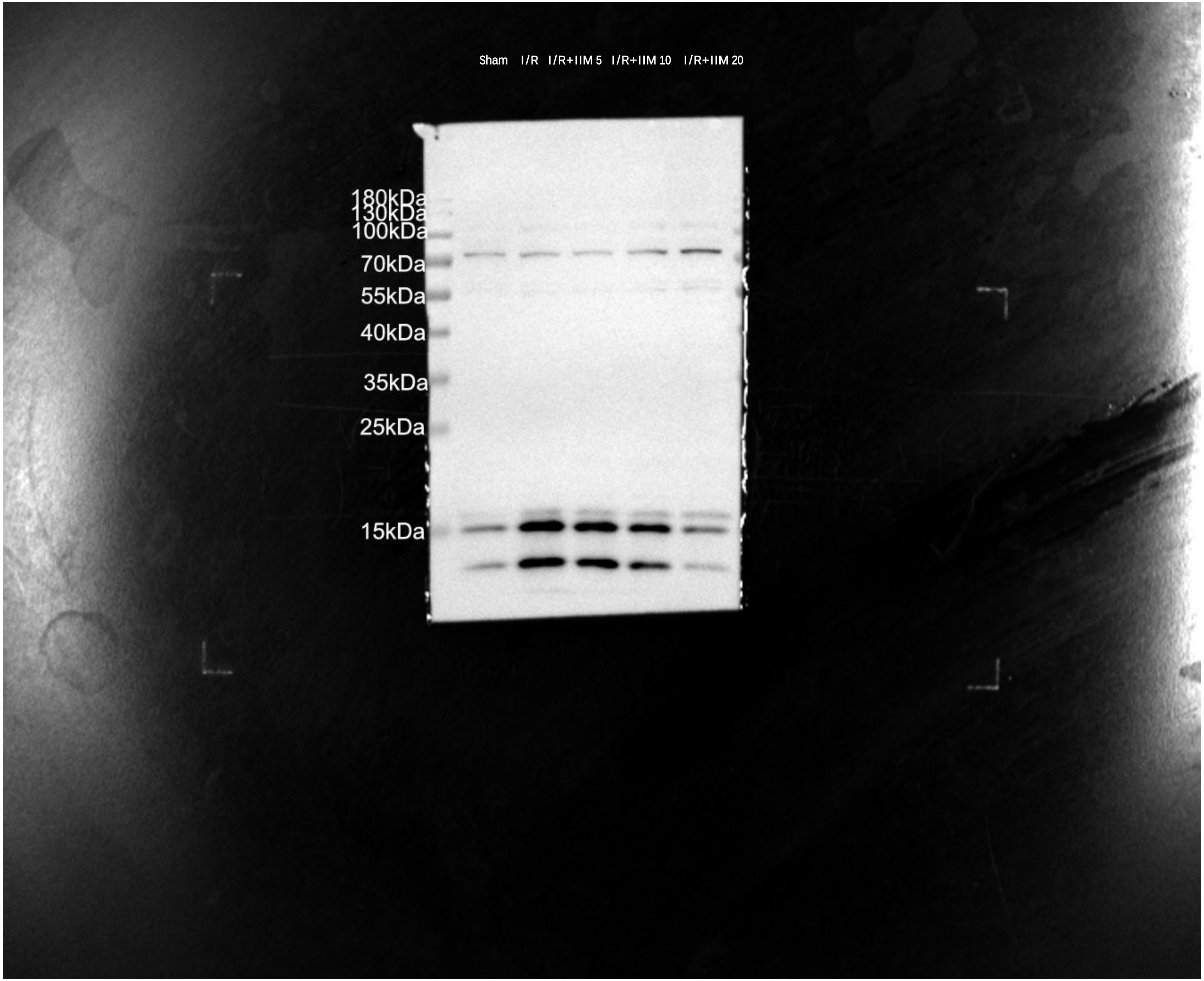

Sham
I/R
I/R+IIM 5
I/R+IIM 10
I/R+IIM 20
180kDa
130kDa
100kDa
70kDa
55kDa
40kDa
35kDa
25kDa
15kDa

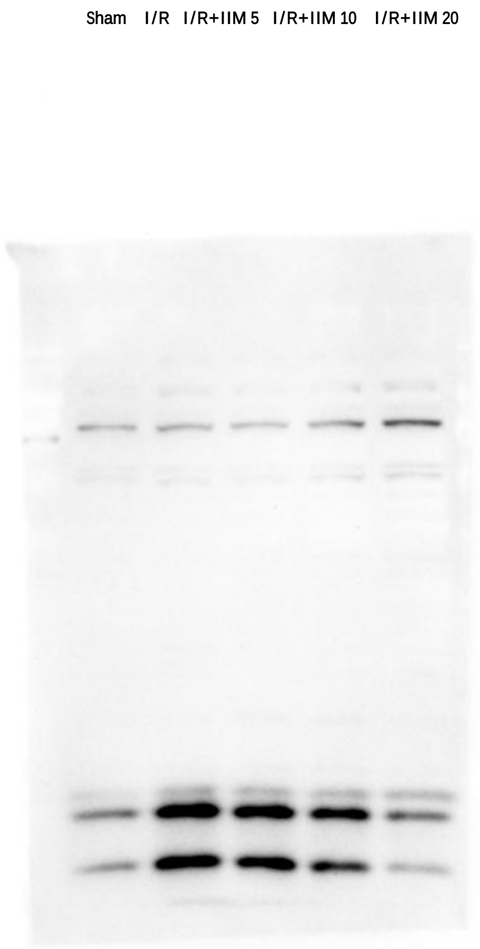
Sham
I/R
I/R+IIM 5
I/R+IIM 10
I/R+IIM 20

Fig2C ACTIN

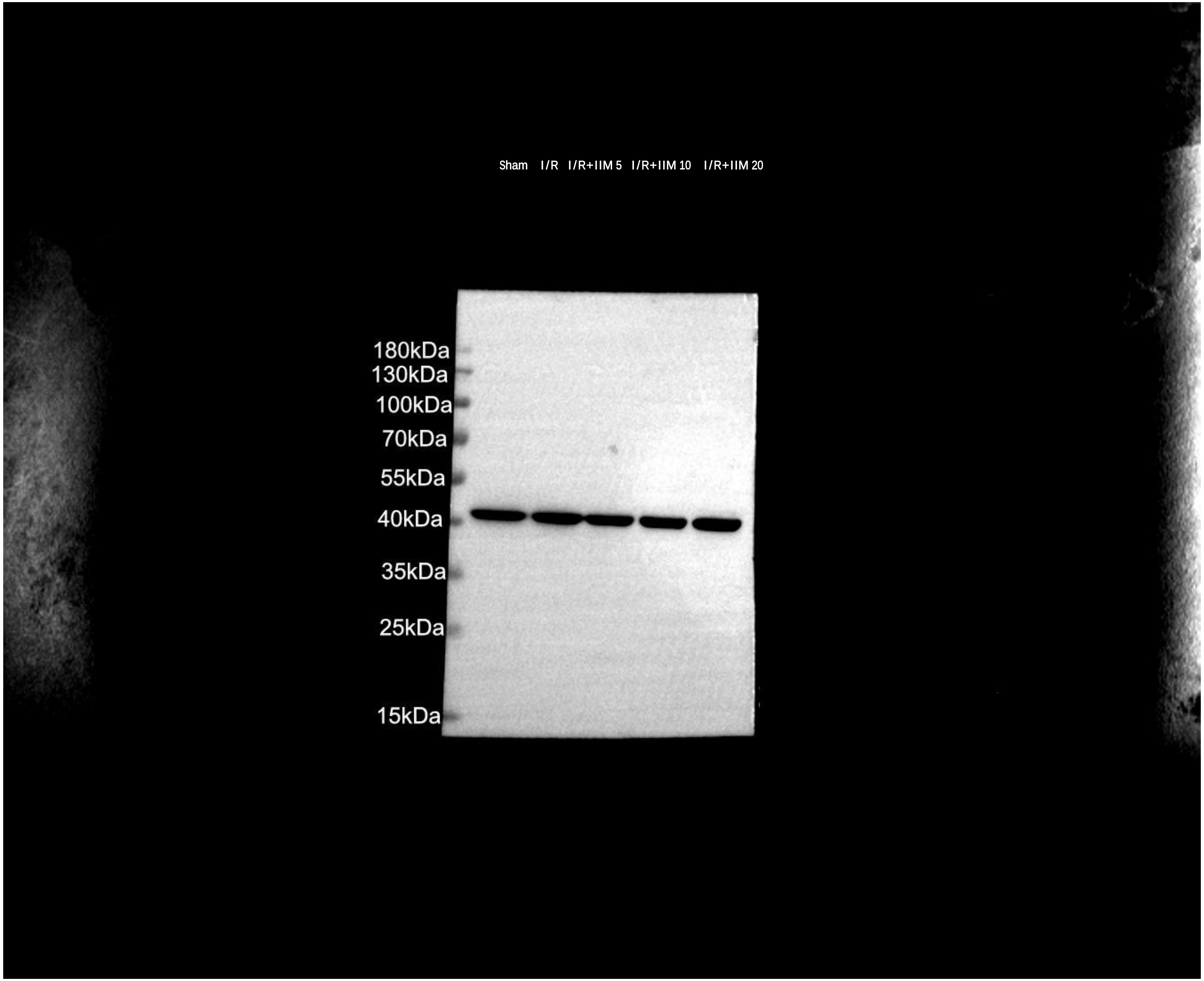

Sham
I/R
I/R+IIM 5
I/R+IIM 10
I/R+IIM 20
180kDa
130kDa
100kDa
70kDa
55kDa
40kDa
35kDa
25kDa
15kDa

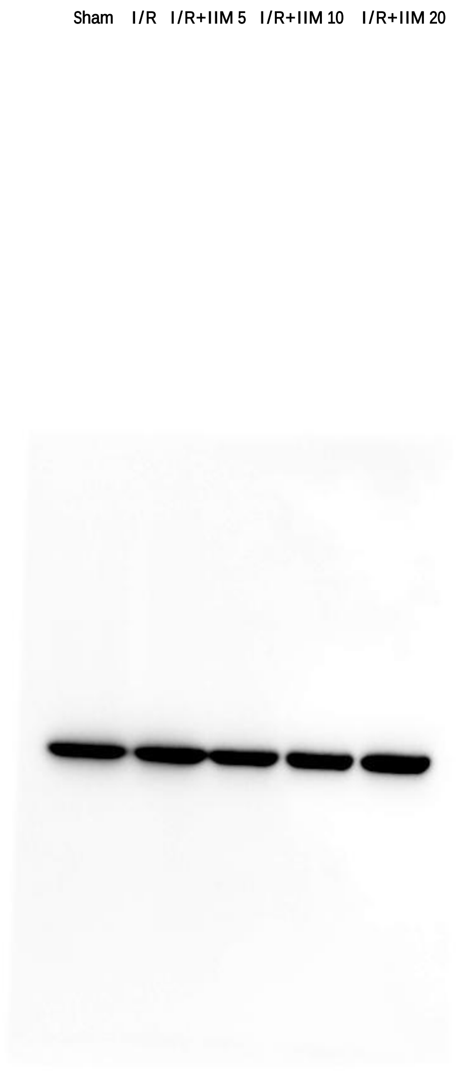
Sham
I/R
I/R+IIM 5
I/R+IIM 10
I/R+IIM 20

Fig3D caspase3

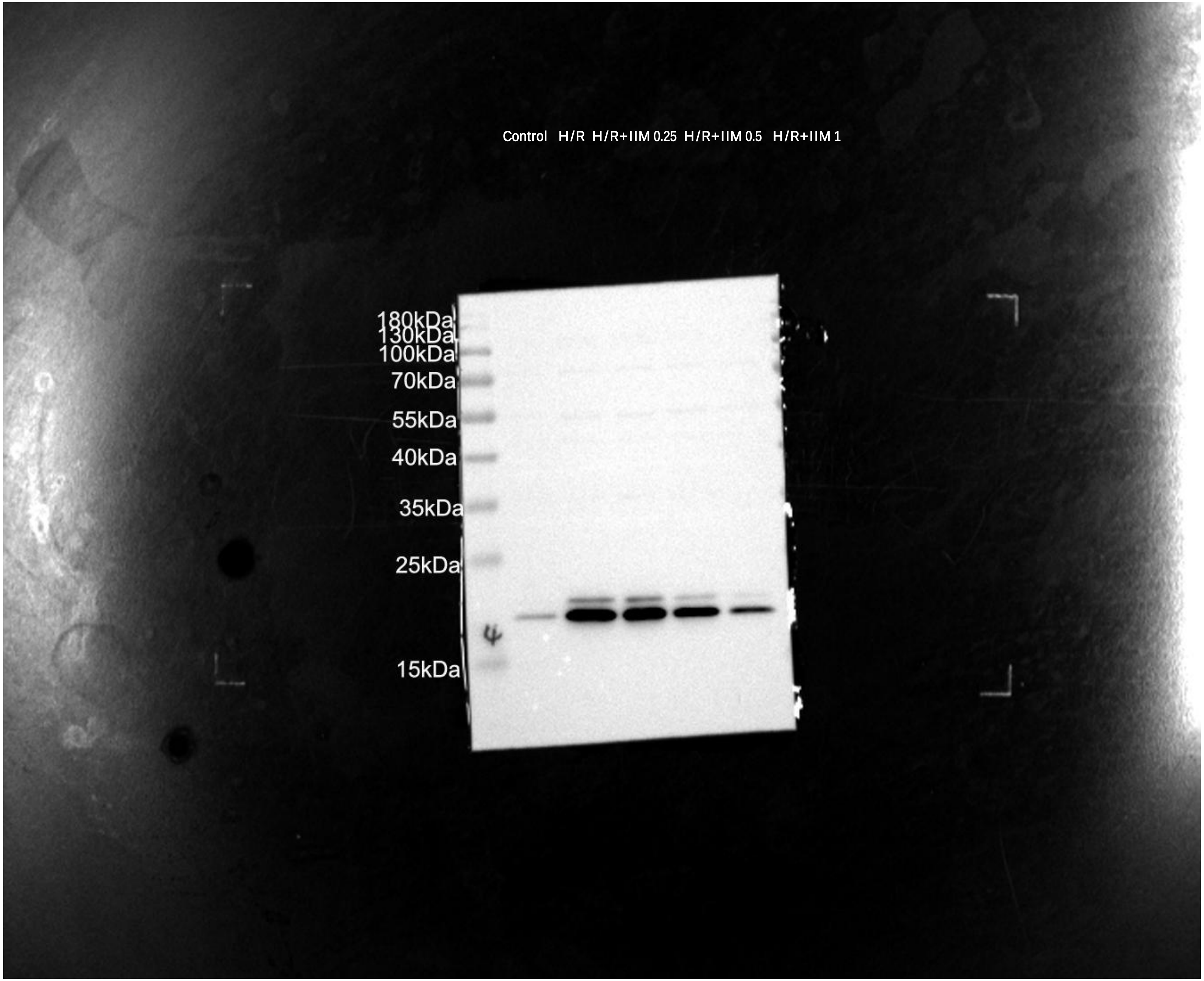

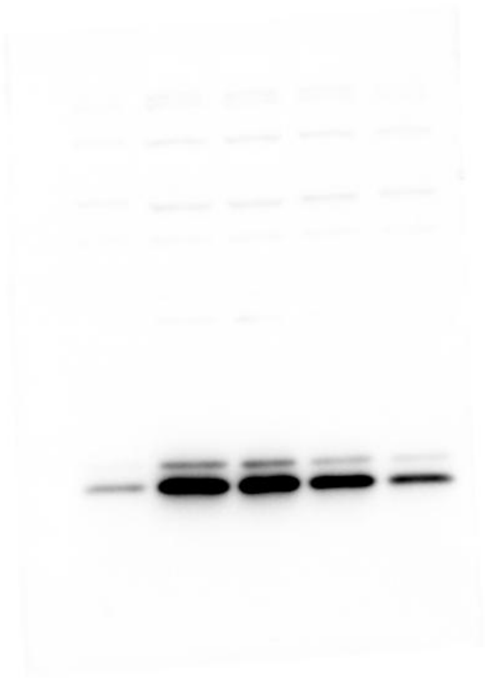
Control
H/R
H/R+IIM 0.25
H/R+IIM 0.5
H/R+IIM 1

Fig3D BAX

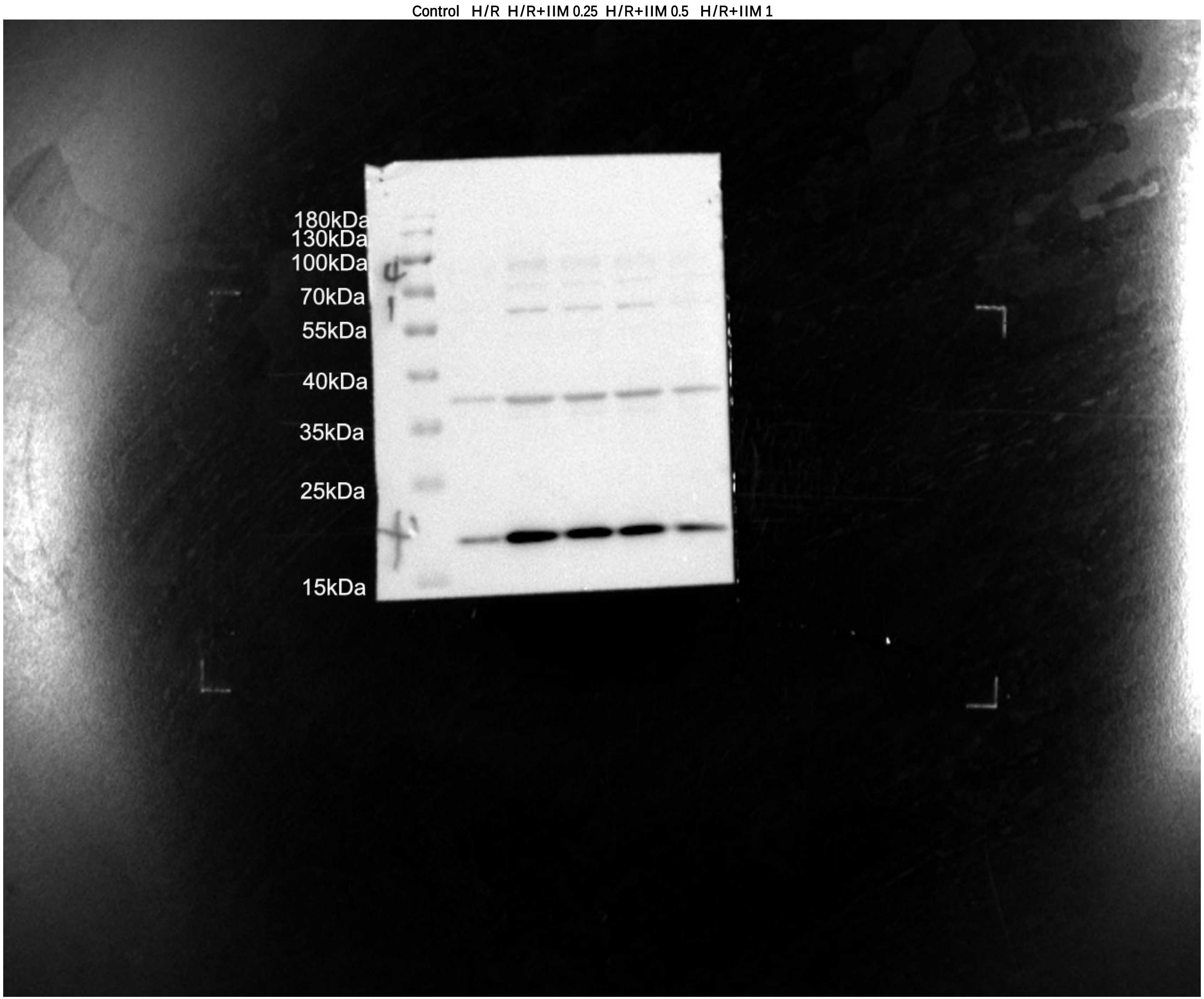
Control H/R H/R+IIM 0.25 H/R+IIM 0.5 H/R+IIM 1
180kDa
130kDa
100kDa
70kDa
55kDa
40kDa
35kDa
25kDa
15kDa

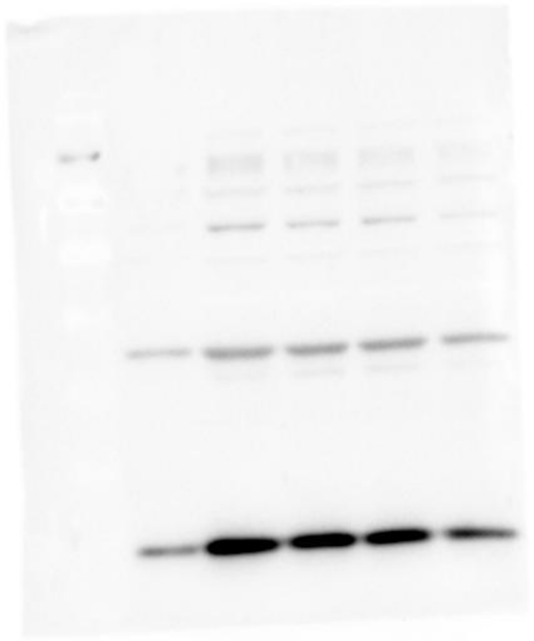
Control
H/R
H/R+IIM 0.25
H/R+IIM 0.5
H/R+IIM 1

Fig3D ACTIN

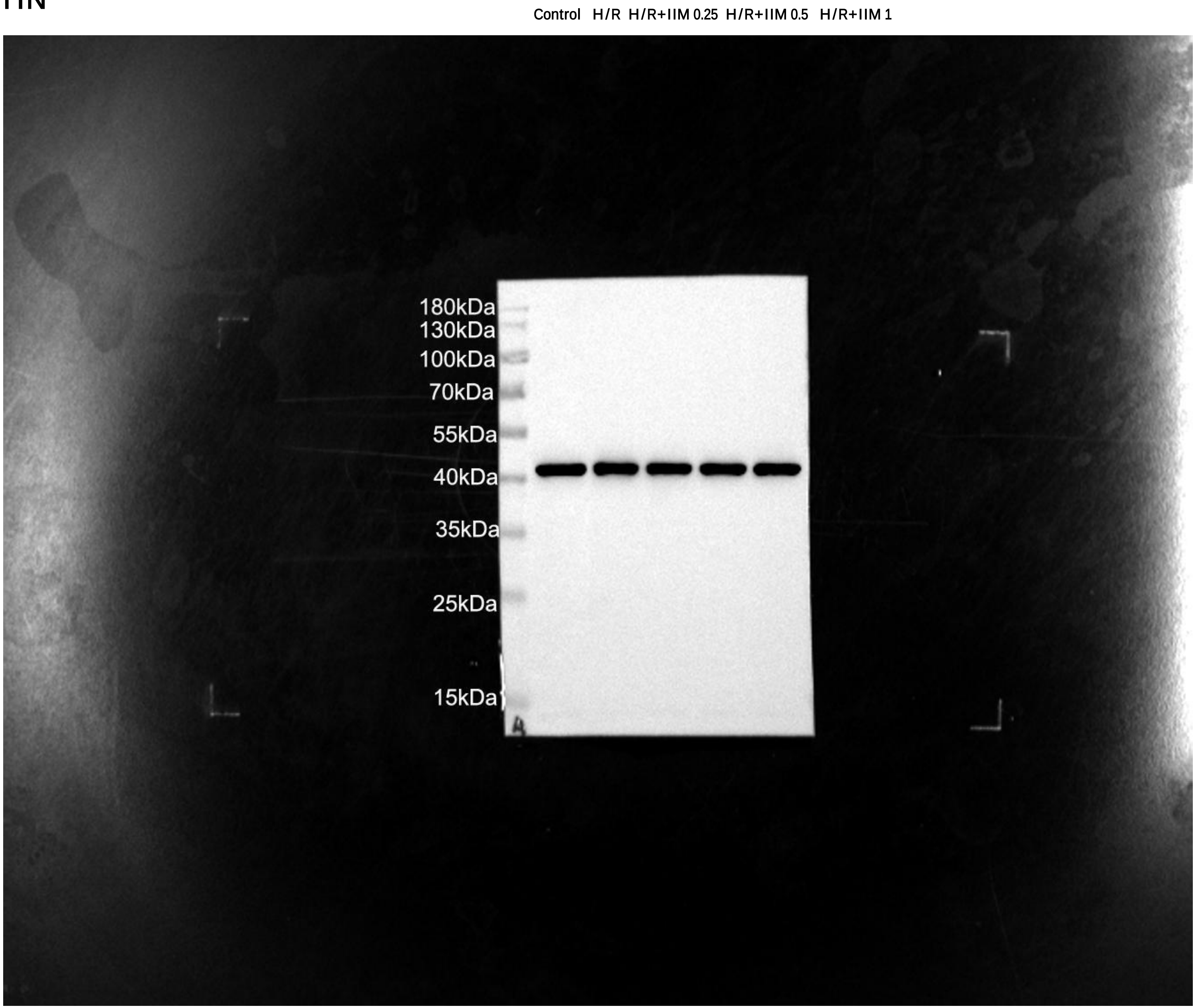

Control
H/R
H/R+IIM 0.25
H/R+IIM 0.5
H/R+IIM 1
180kDa
130kDa
100kDa
70kDa
55kDa
40kDa
35kDa
25kDa
15kDa

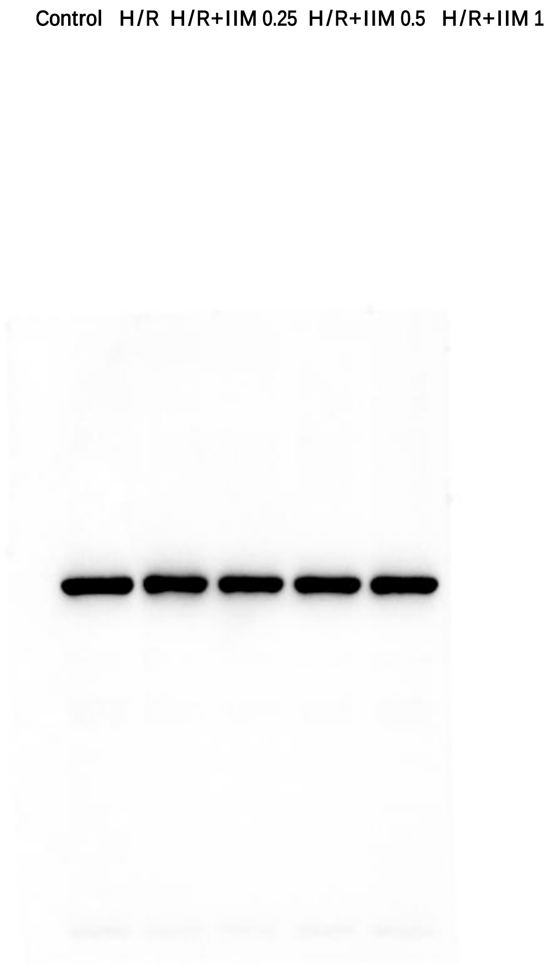
Control
H/R
H/R+IIM 0.25
H/R+IIM 0.5
H/R+IIM 1

Fig3D Bcl2

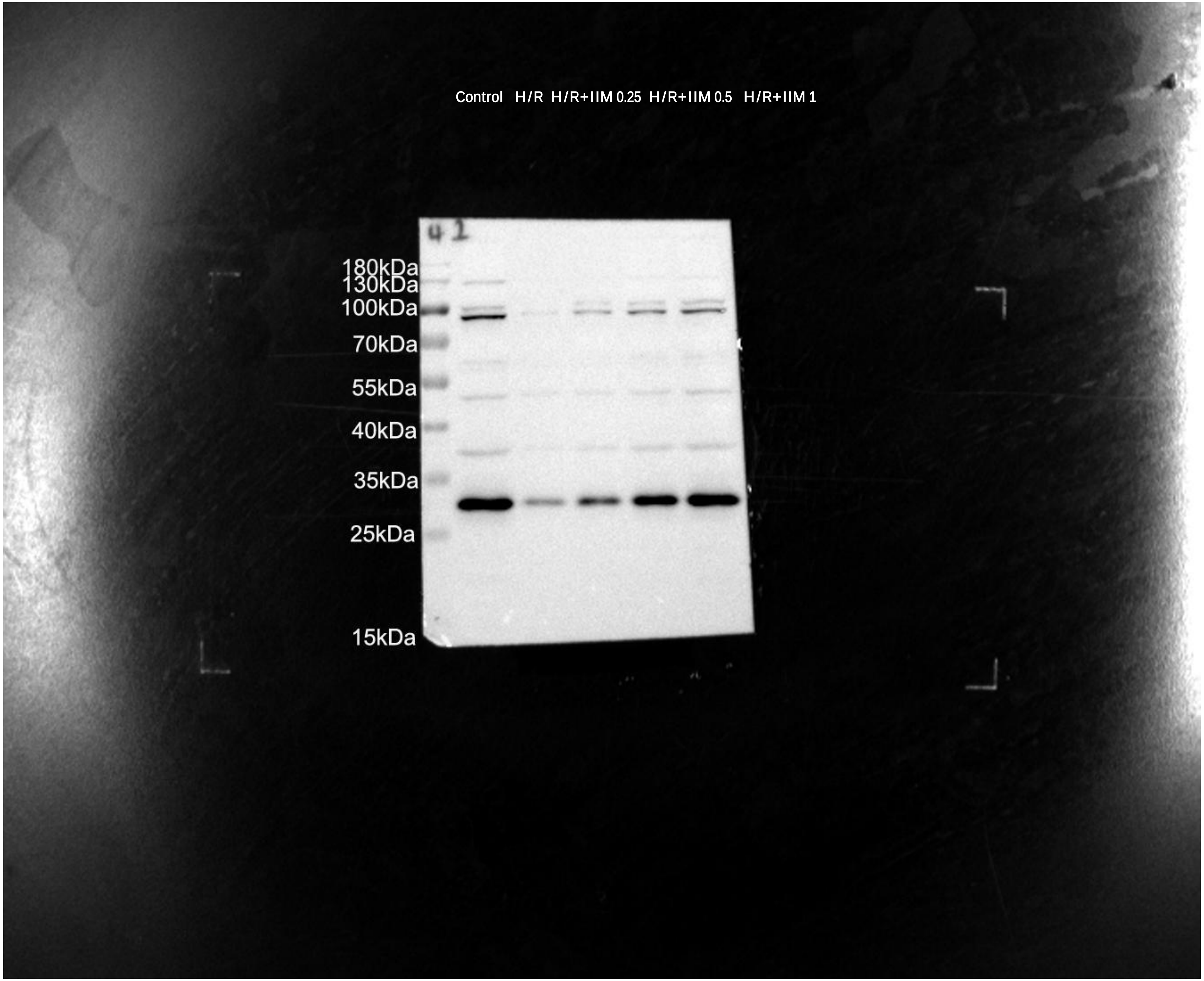
Control H/R H/R+IIM 0.25 H/R+IIM 0.5 H/R+IIM 1
180kDa
130kDa
100kDa
70kDa
55kDa
40kDa
35kDa
25kDa
15kDa

Control H/R H/R+IIM 0.25 H/R+IIM 0.5 H/R+IIM 1

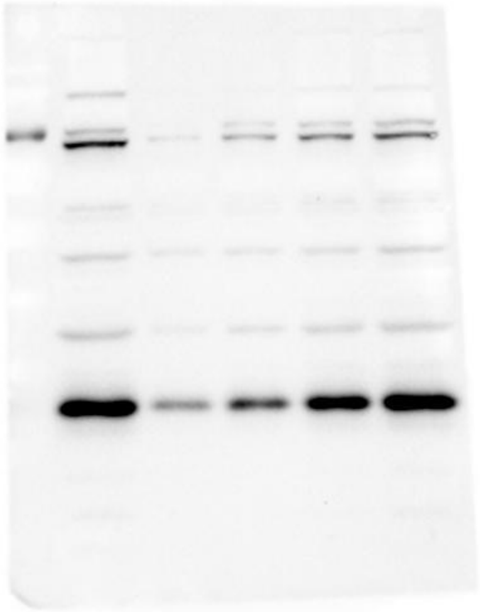

Fig4A actin

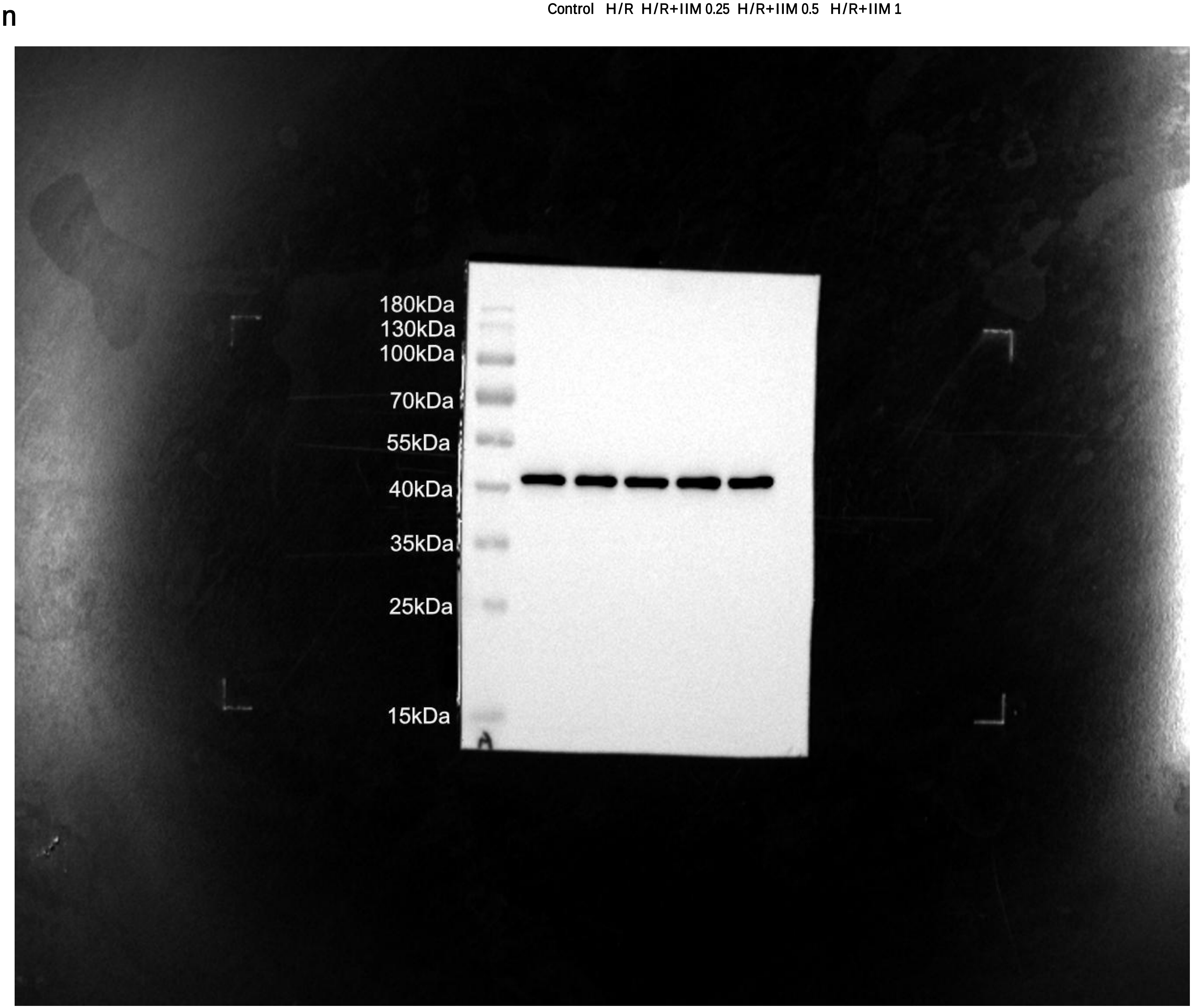
Control H/R H/R+IIM 0.25 H/R+IIM 0.5 H/R+IIM 1
180kDa
130kDa
100kDa
70kDa
55kDa
40kDa
35kDa
25kDa
15kDa

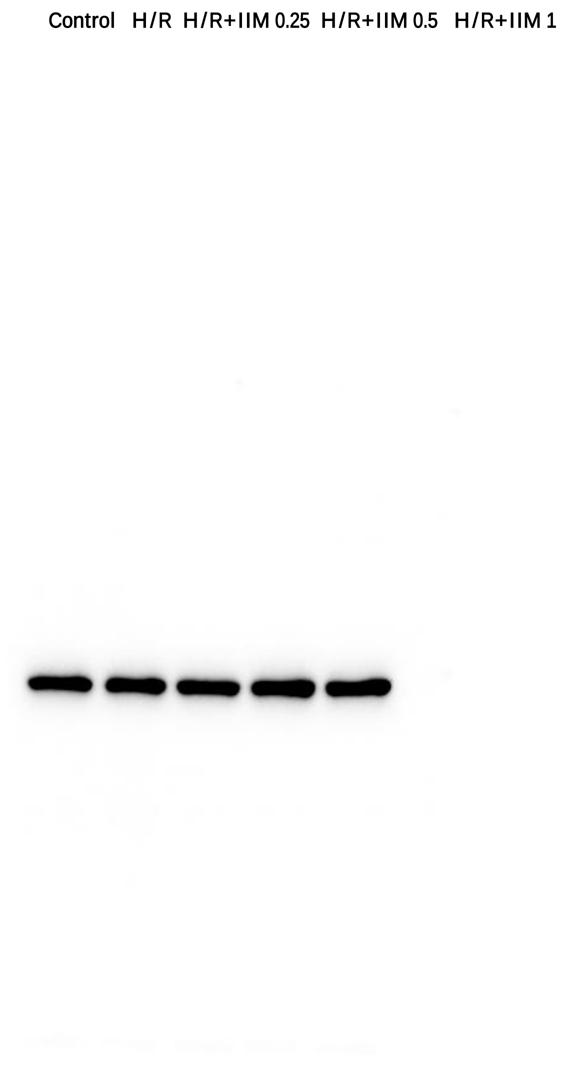
Control
H/R
H/R+IIM 0.25
H/R+IIM 0.5
H/R+IIM 1

Fig4A Beclin1

Control H/R H/R+IIM 0.25 H/R+IIM 0.5 H/R+IIM 1

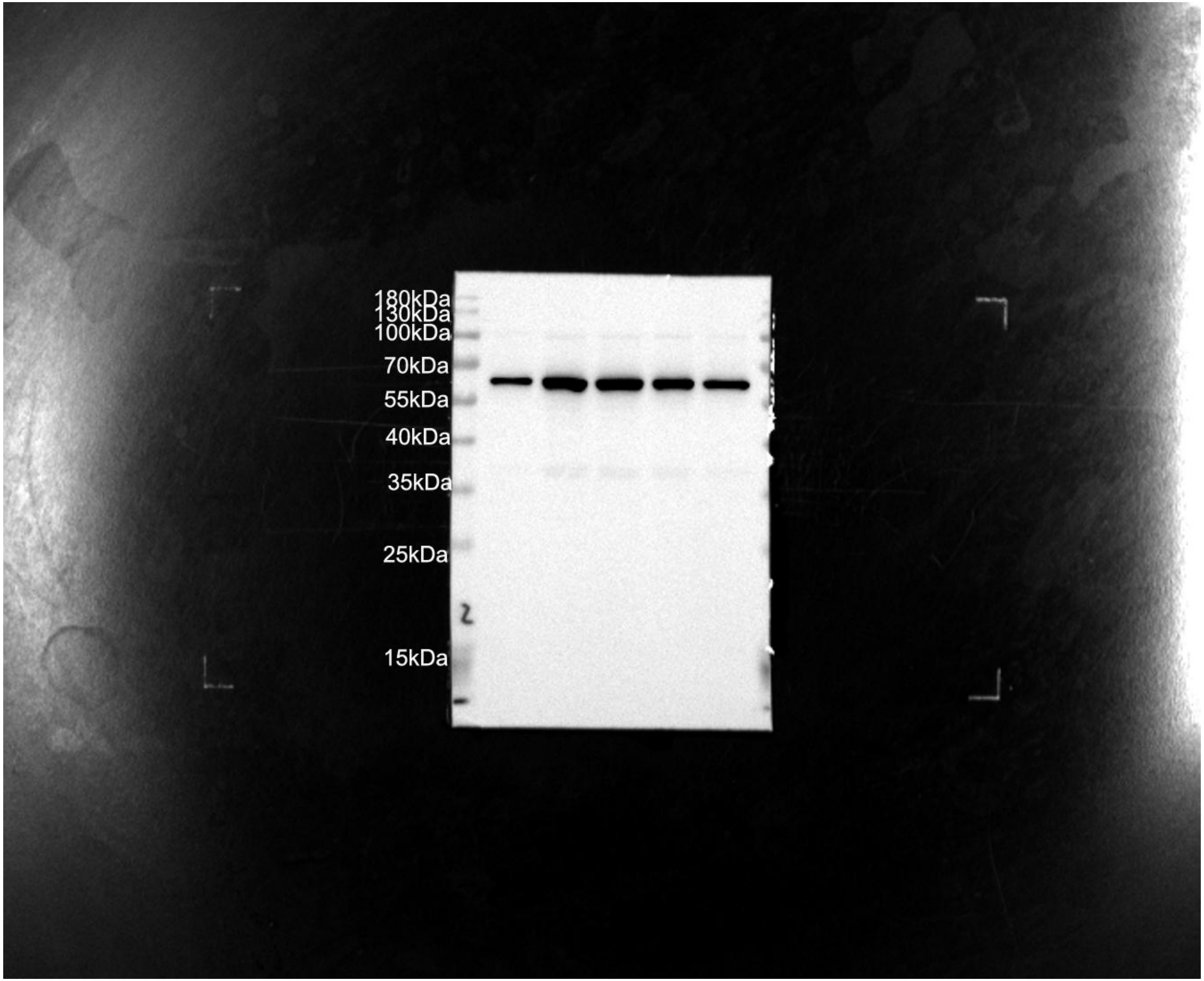

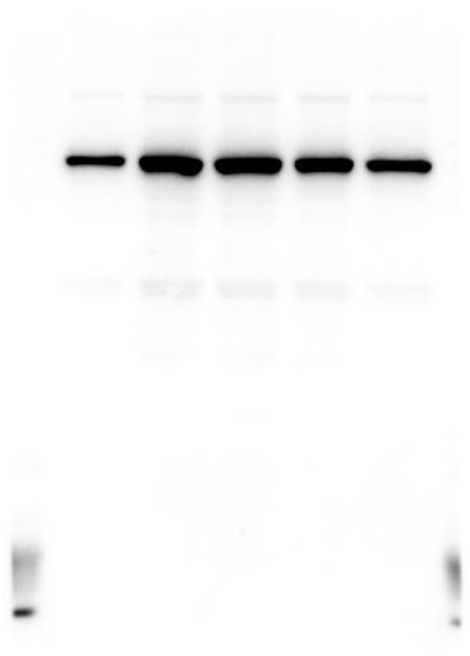
Control
H/R
H/R+IIM 0.25
H/R+IIM 0.5
H/R+IIM 1

Fig4A LC3

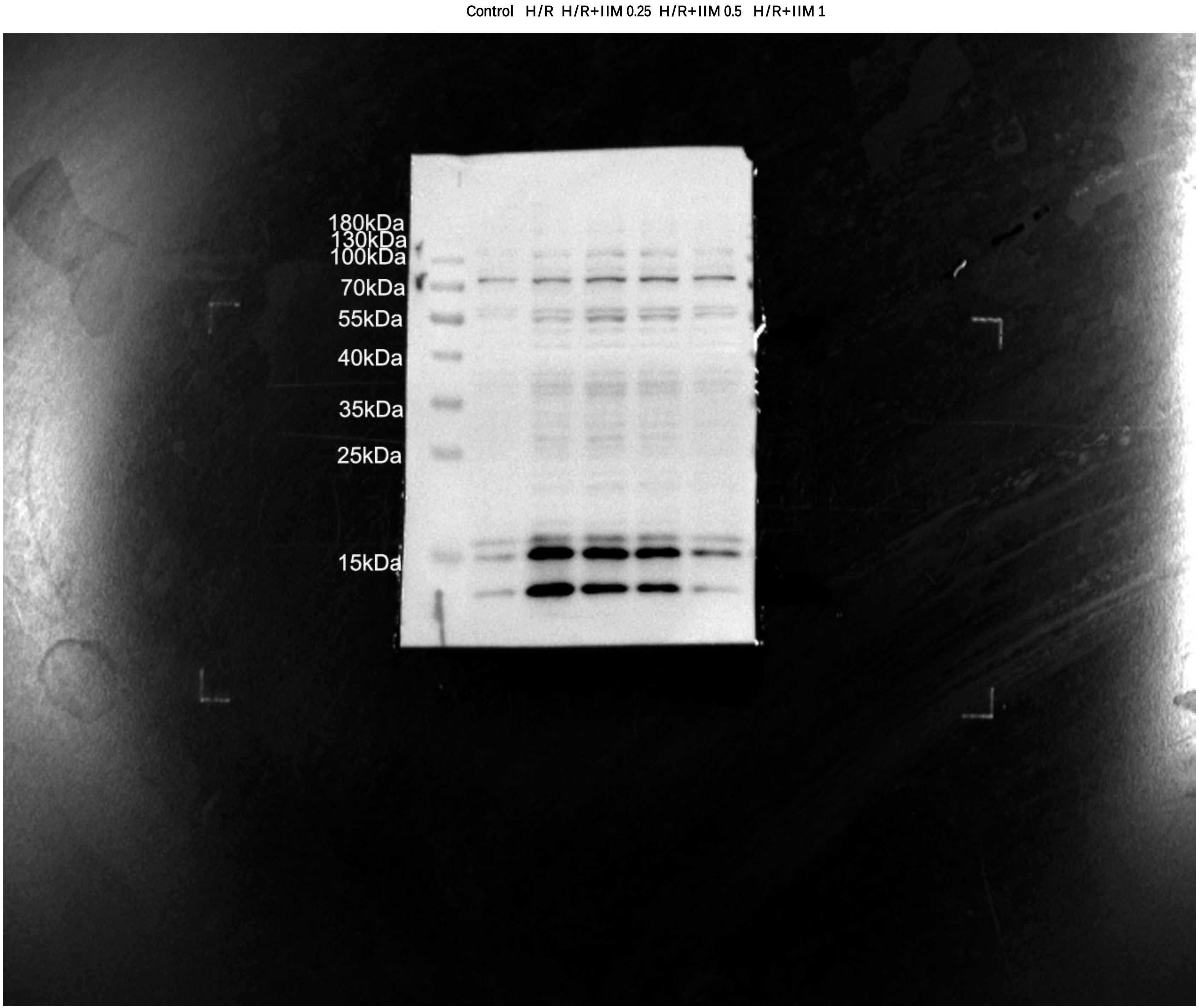

Control H/R H/R+IIM 0.25 H/R+IIM 0.5 H/R+IIM 1
180kDa
130kDa
100kDa
70kDa
55kDa
40kDa
35kDa
25kDa
15kDa

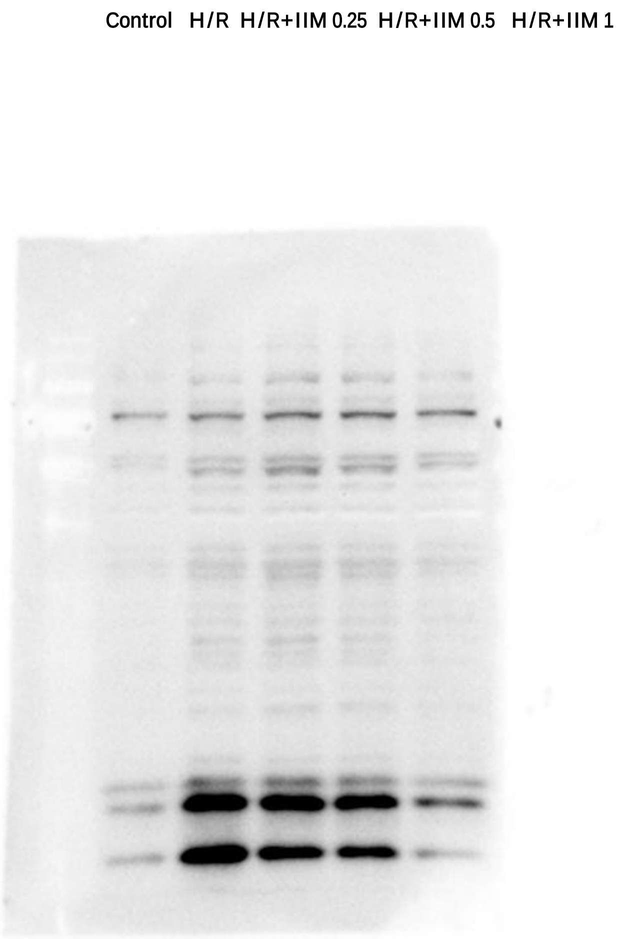
Control H/R H/R+IIM 0.25 H/R+IIM 0.5 H/R+IIM 1

Fig5B ACTIN

Control H/R H/R+IIM 0.25 H/R+IIM 0.5 H/R+IIM 1

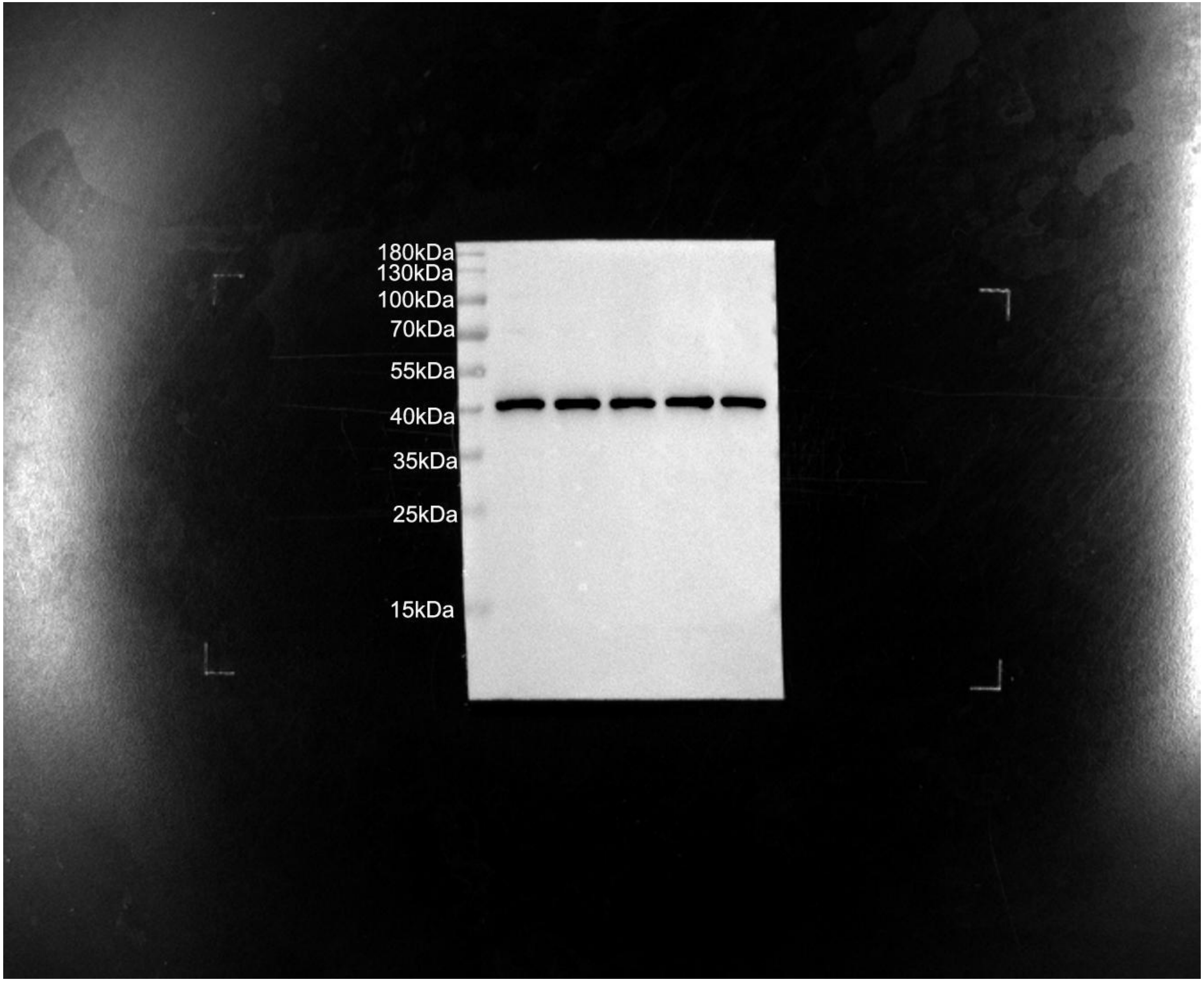

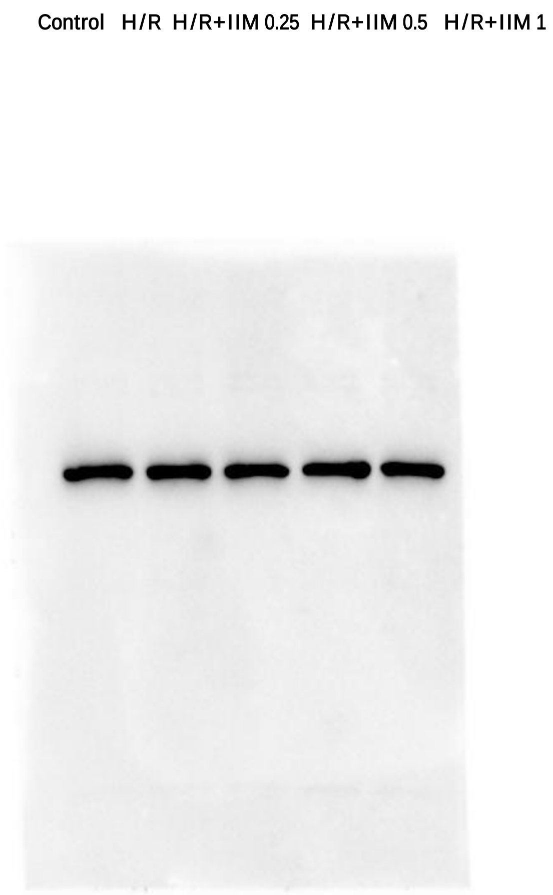
Control
H/R
H/R+IIM 0.25
H/R+IIM 0.5
H/R+IIM 1

Fig5B NFkB

Control H/R H/R+IIM 0.25 H/R+IIM 0.5 H/R+IIM 1

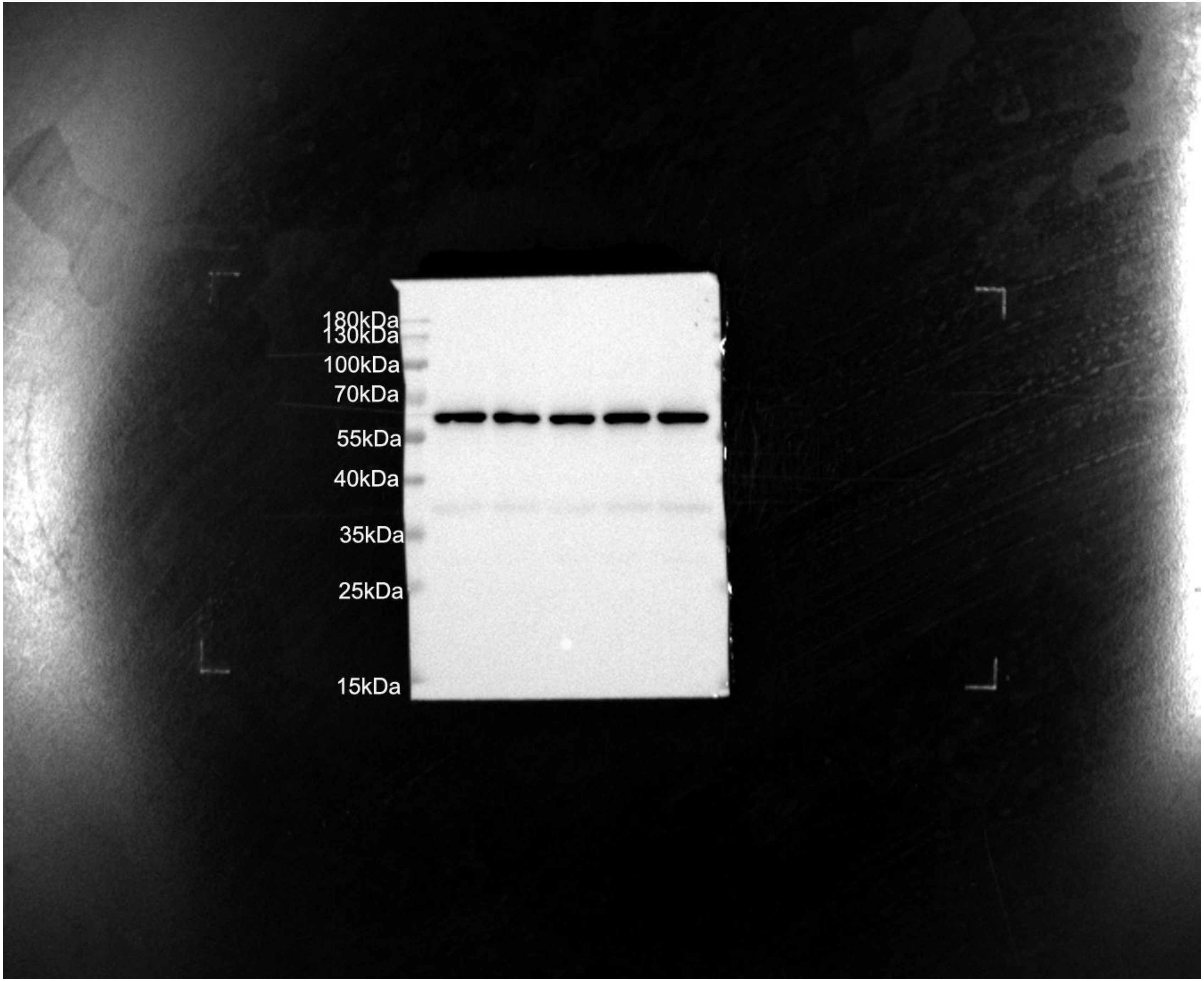

Control H/R H/R+IIM 0.25 H/R+IIM 0.5 H/R+IIM 1

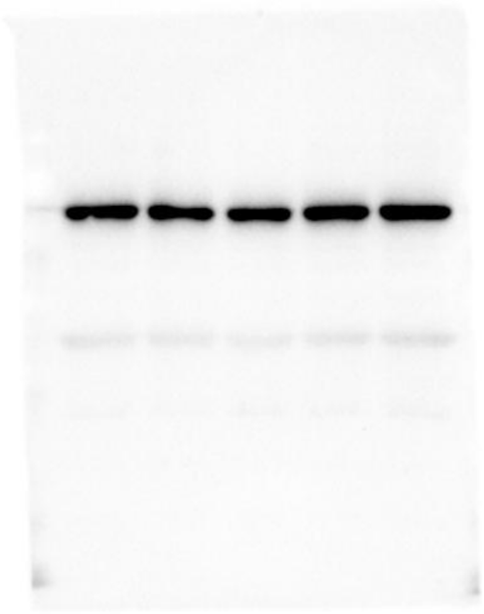

Fig5B p-NFkB

Control H/R H/R+IIM 0.25 H/R+IIM 0.5 H/R+IIM 1

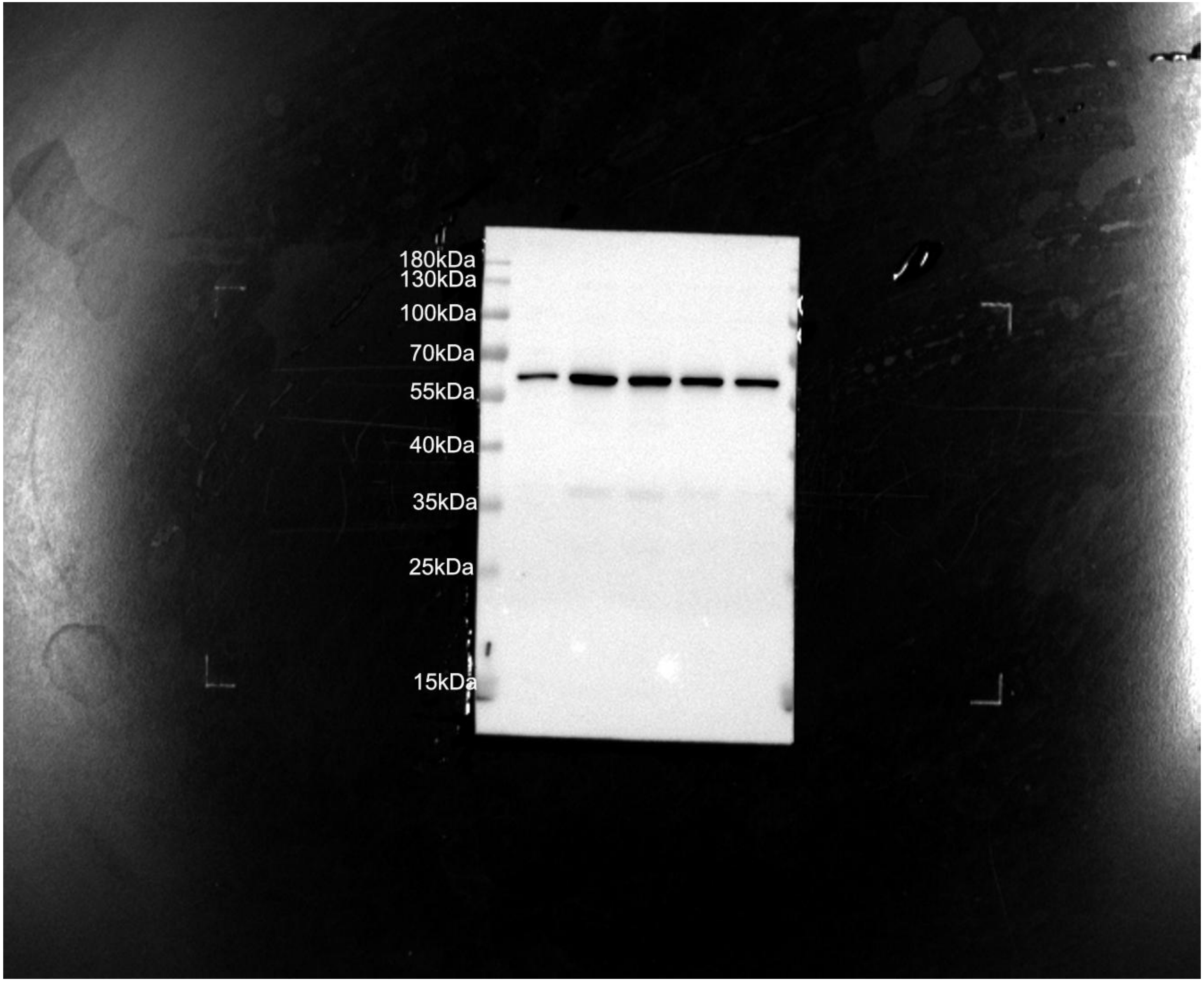

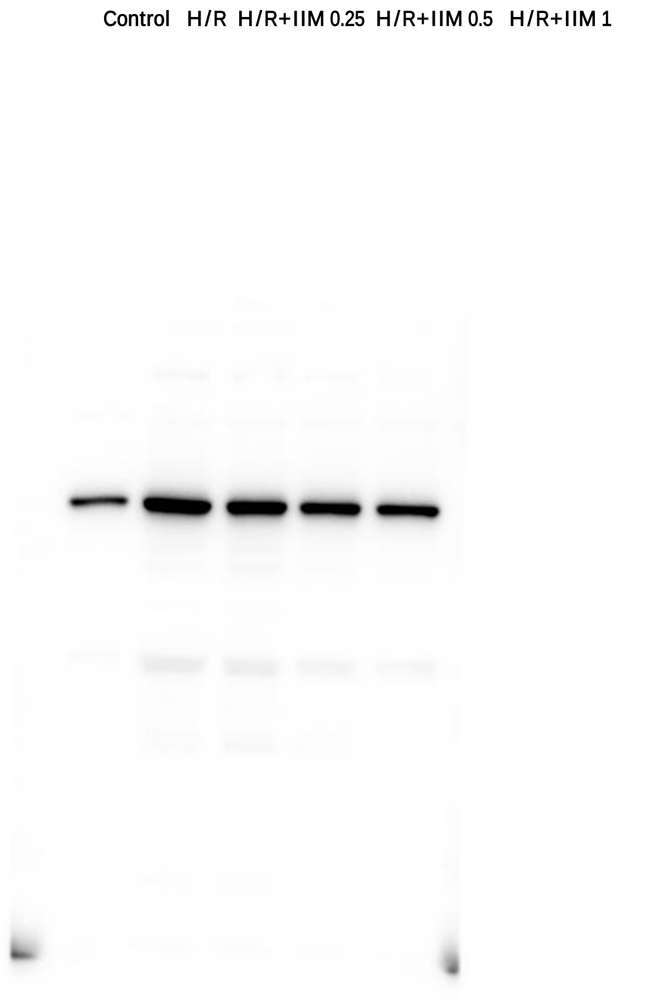
Control
H/R
H/R+IIM 0.25
H/R+IIM 0.5
H/R+IIM 1

Fig5B KLF4

Control H/R H/R+IIM 0.25 H/R+IIM 0.5 H/R+IIM 1

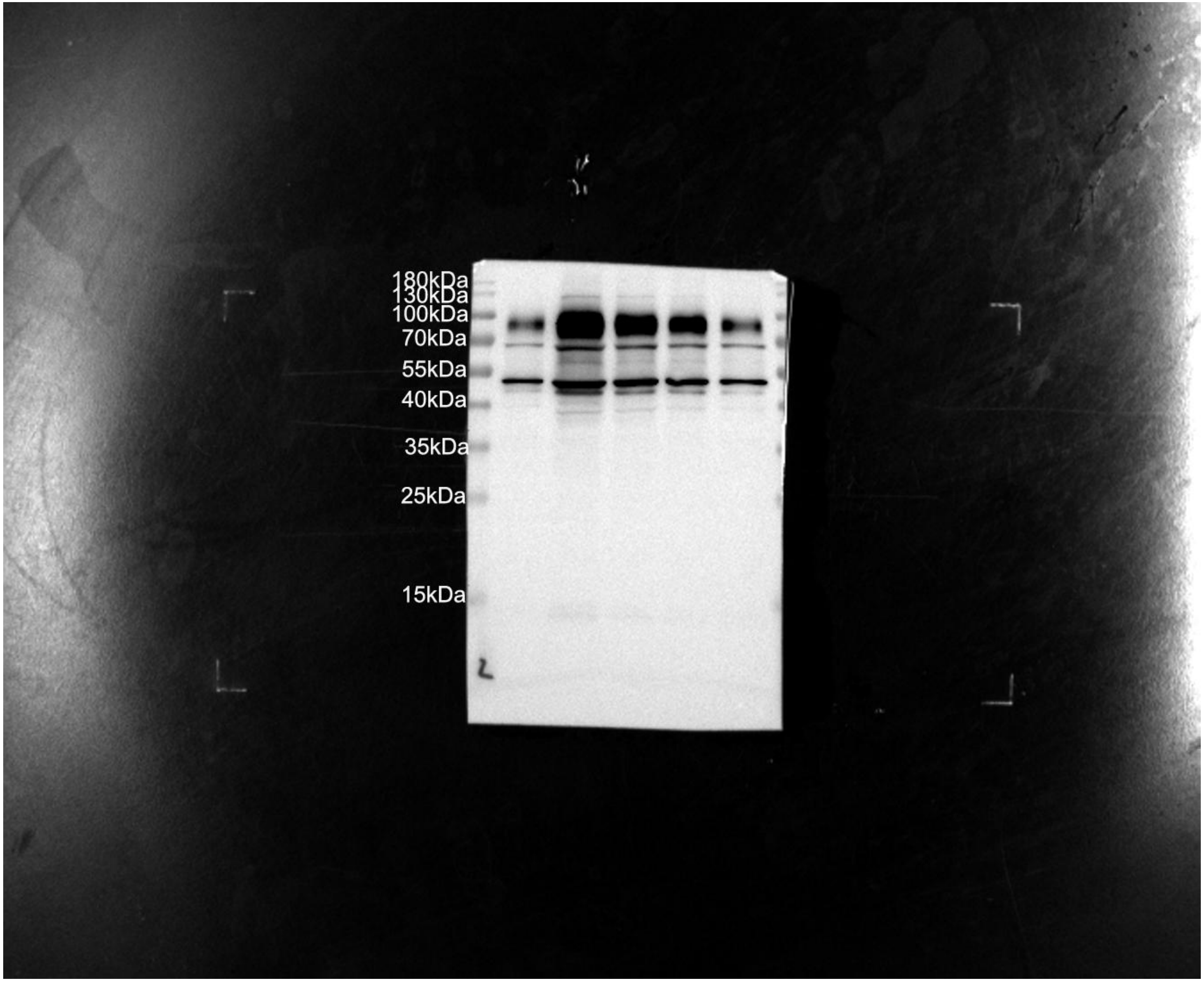

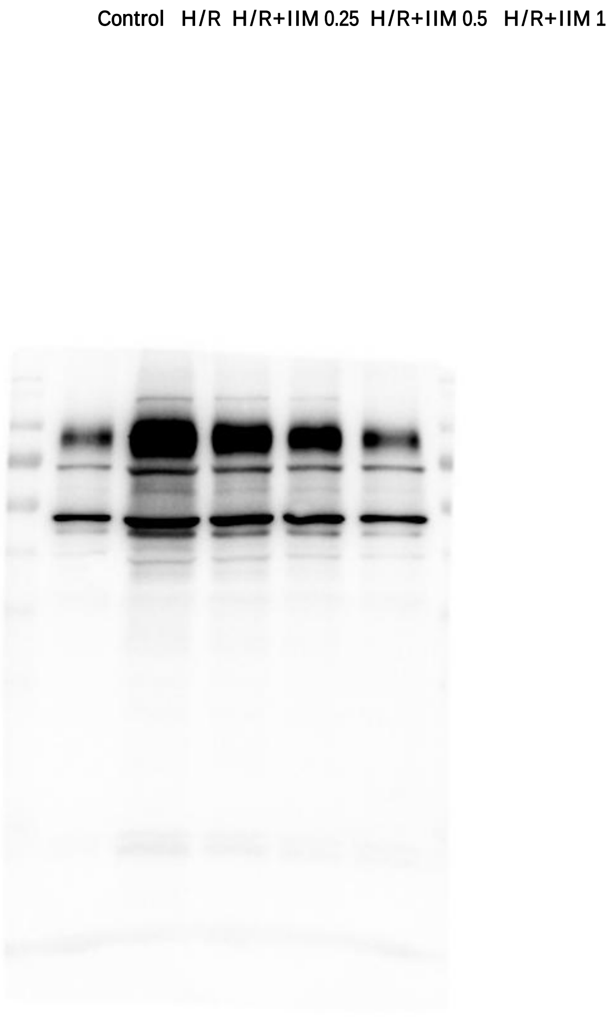
Control
H/R
H/R+IIM 0.25
H/R+IIM 0.5
H/R+IIM 1

Fig5D KLF4

H/R H/R+IIM1 H/R+IIM1 +KLF4

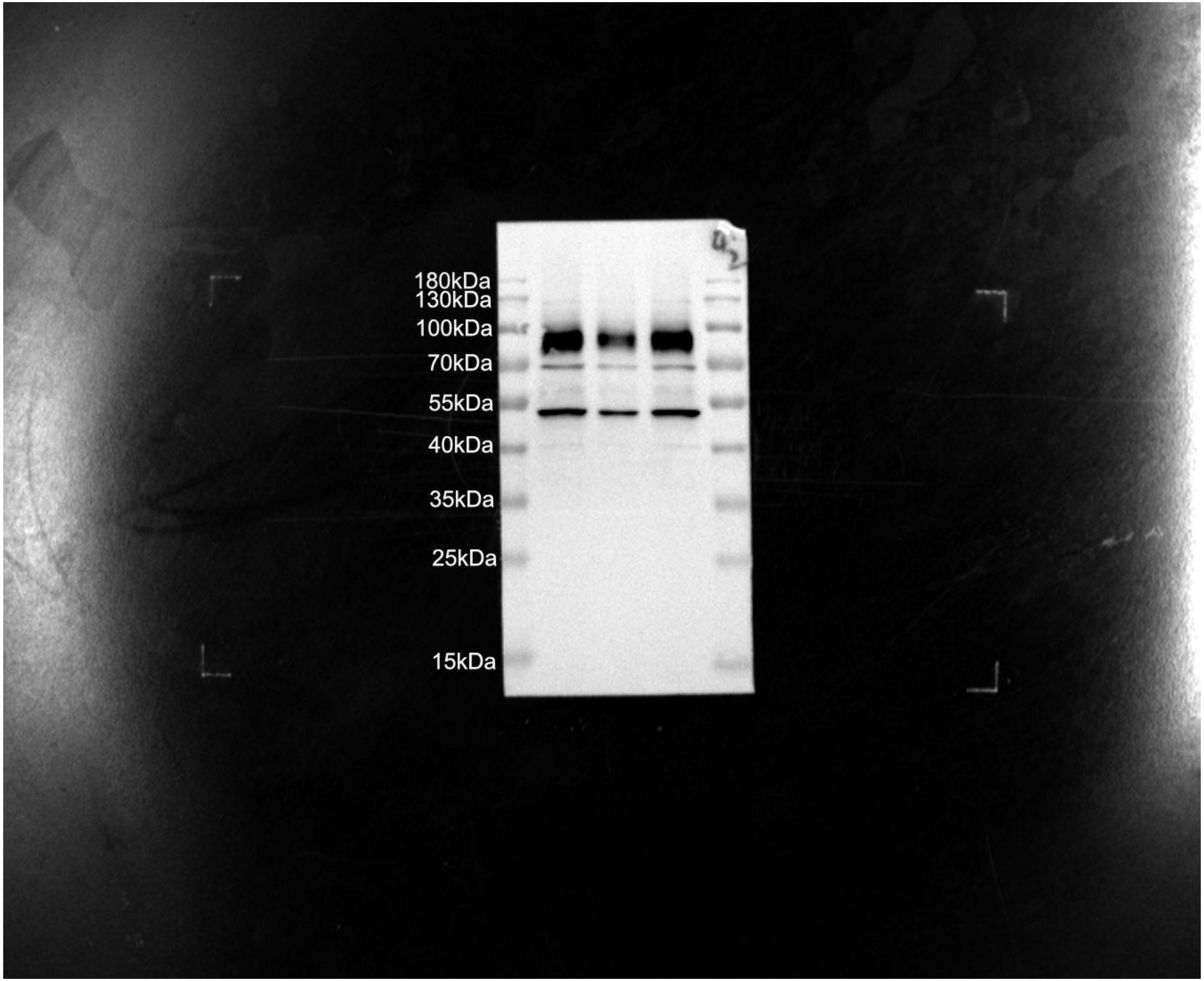

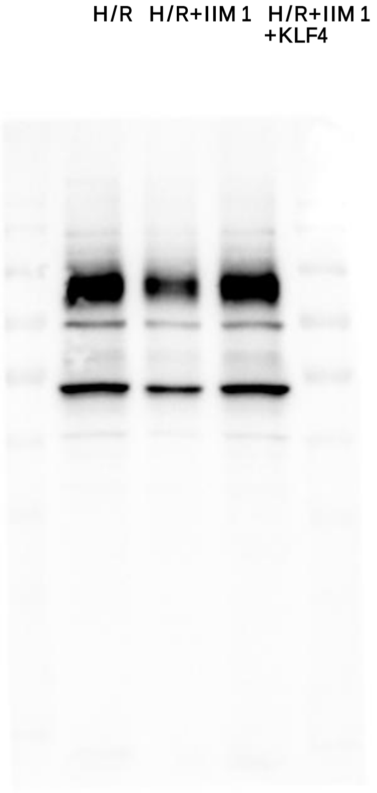

H/R
H/R+IIM 1
H/R+IIM 1
+KLF4

Fig5D NFkB

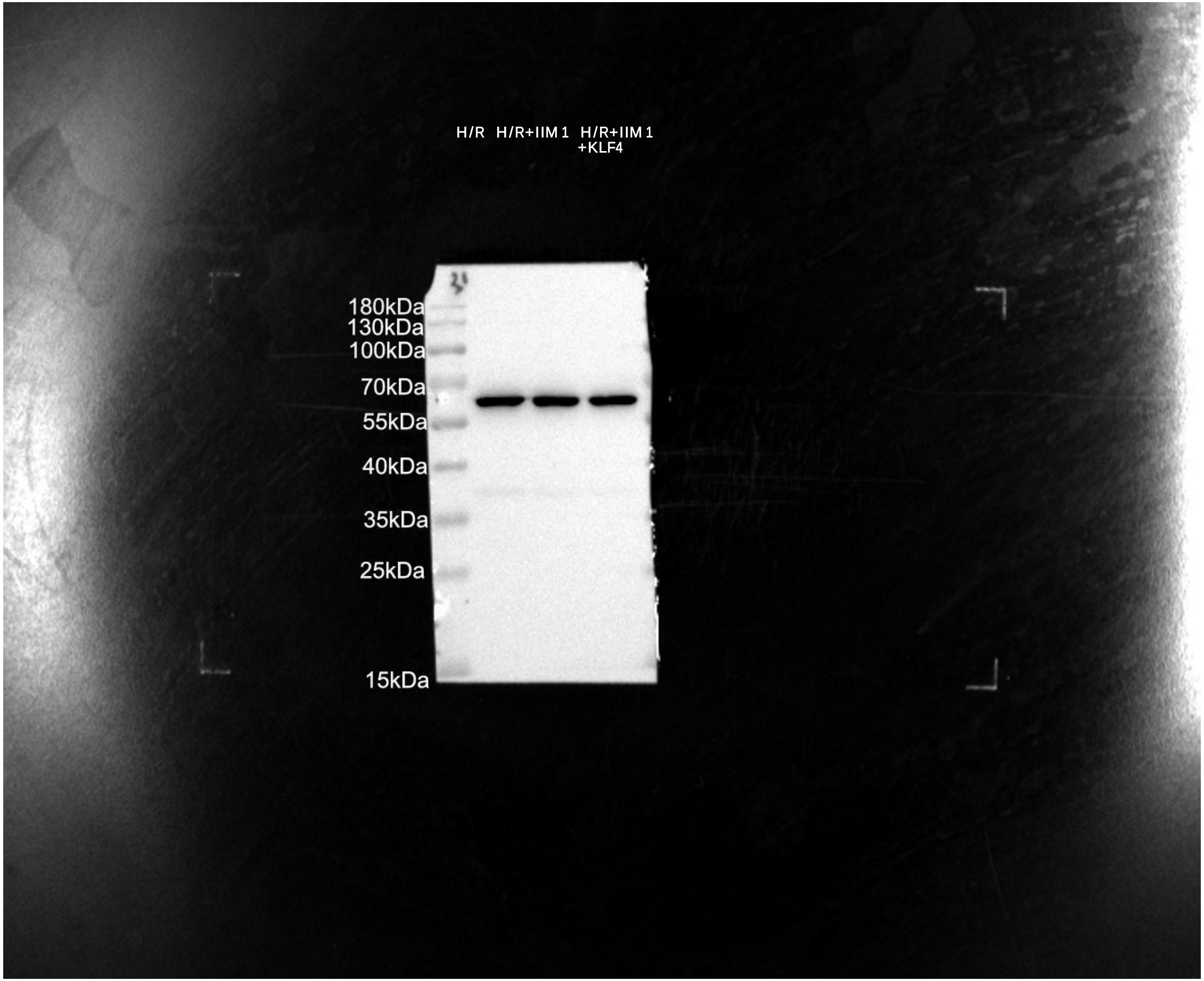
H/R
H/R+IIM 1
H/R+IIM 1 +KLF4
180kDa
130kDa
100kDa
70kDa
55kDa
40kDa
35kDa
25kDa
15kDa

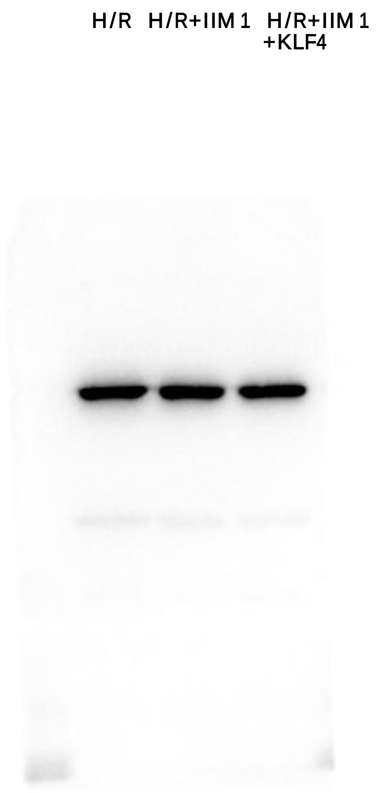
H/R
H/R+IIM 1
H/R+IIM 1
+KLF4

Fig5D p-NFkB

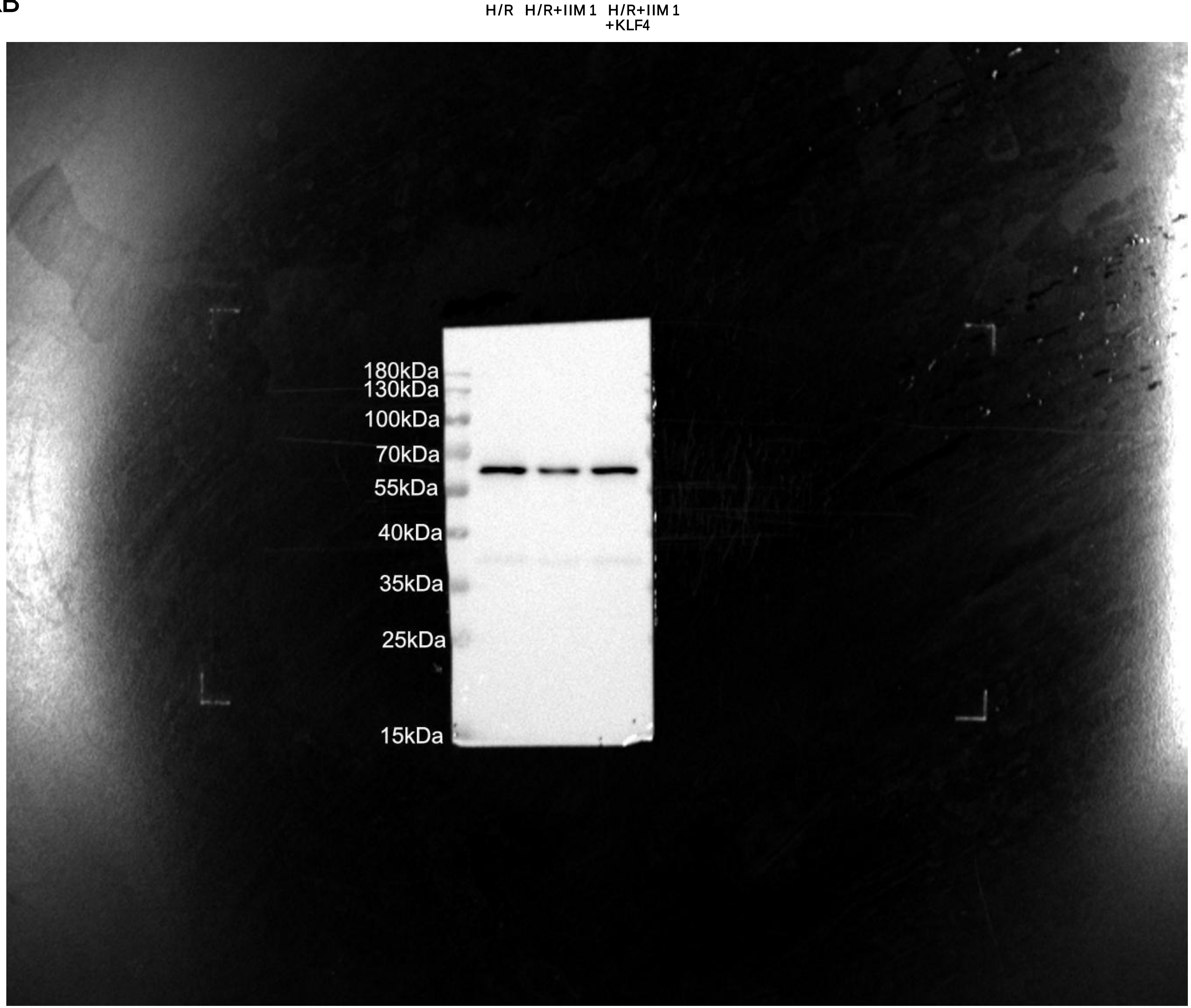
H/R
H/R+IIM1
H/R+IIM1 +KLF4
180kDa
130kDa
100kDa
70kDa
55kDa
40kDa
35kDa
25kDa
15kDa

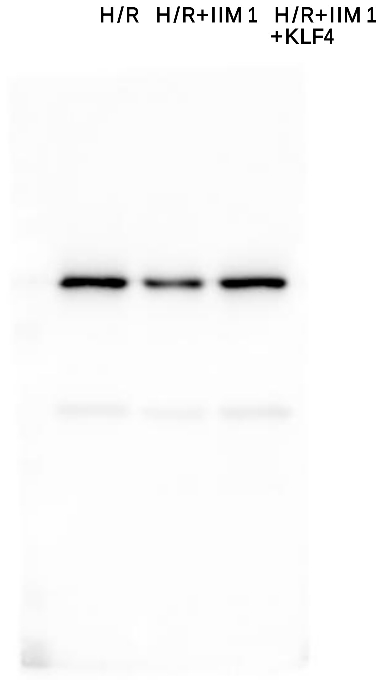
H/R
H/R+IIM 1
H/R+IIM 1
+KLF4

Fig 5D Actin

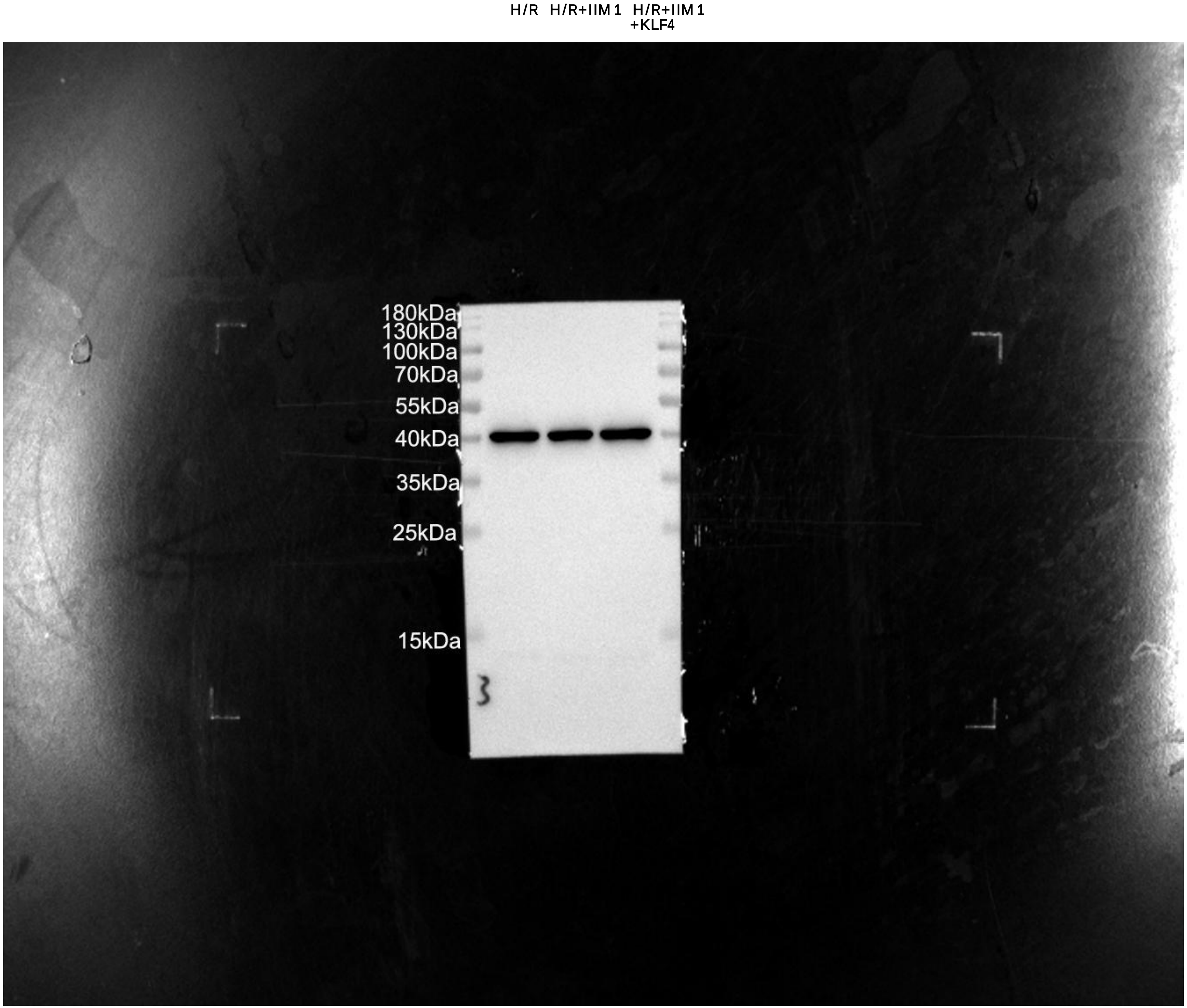

H/R
H/R+IIM 1
H/R+IIM 1 +KLF4
180kDa
130kDa
100kDa
70kDa
55kDa
40kDa
35kDa
25kDa
15kDa
3

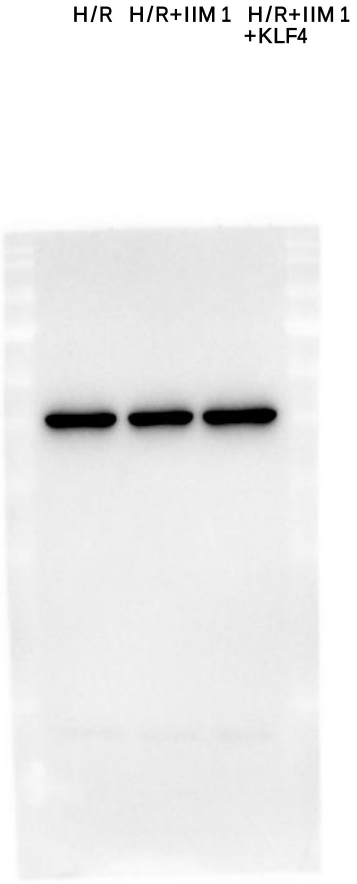
H/R
H/R+IIM1
H/R+IIM1 +KLF4

Fig6D ACTIN

H/R H/R+IIM 1 H/R+IIM 1 +KLF4

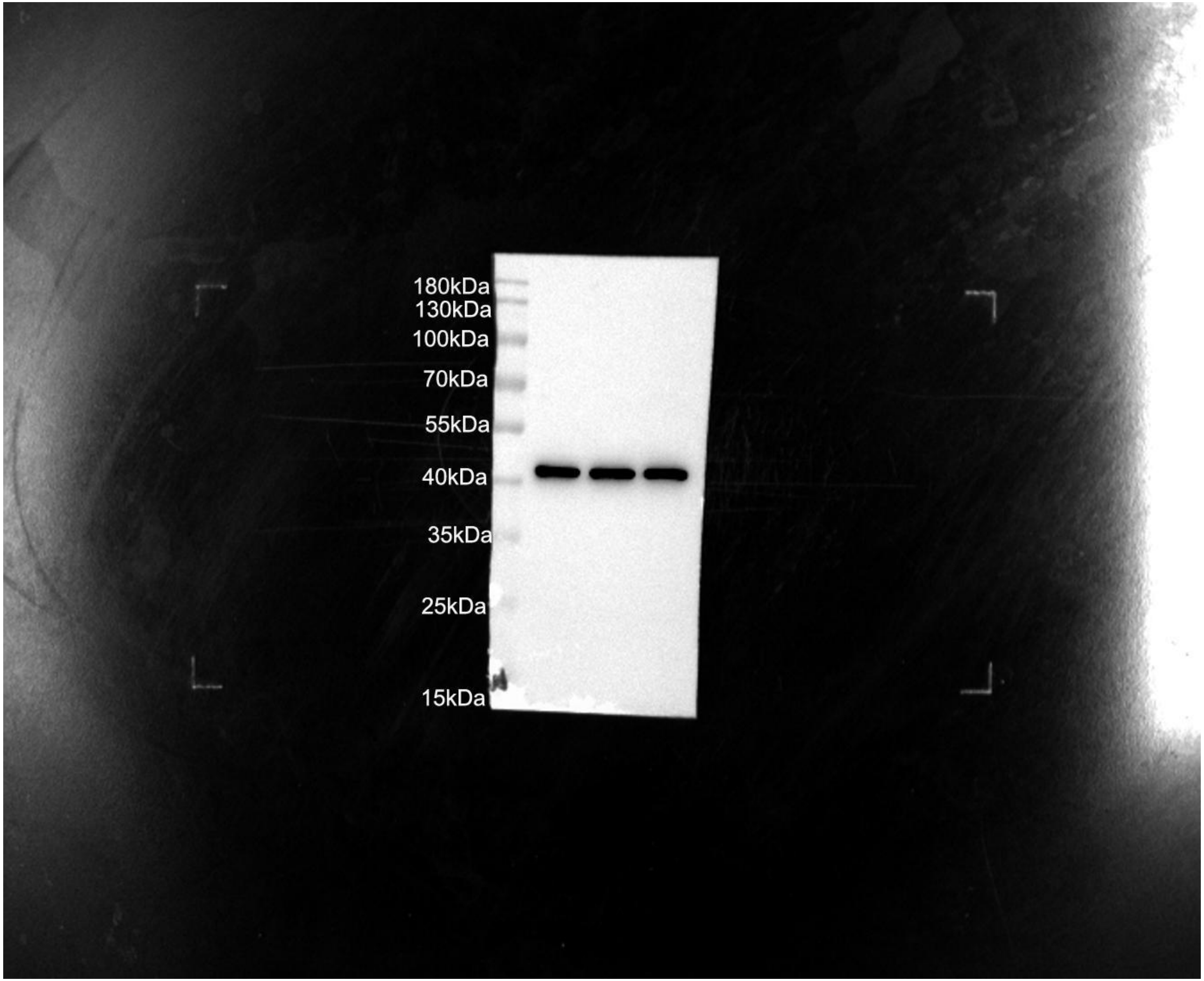

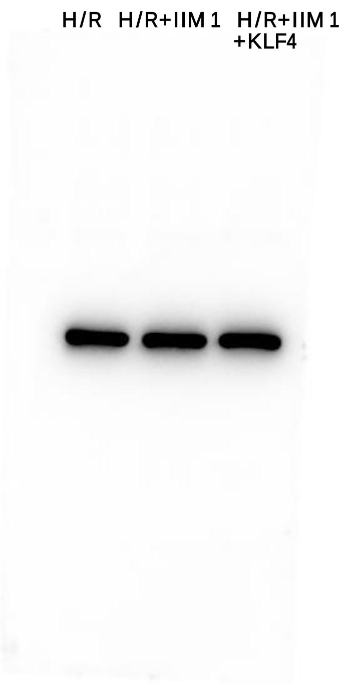
H/R
H/R+IIM 1
H/R+IIM 1
+KLF4

Fig6D Beclin1

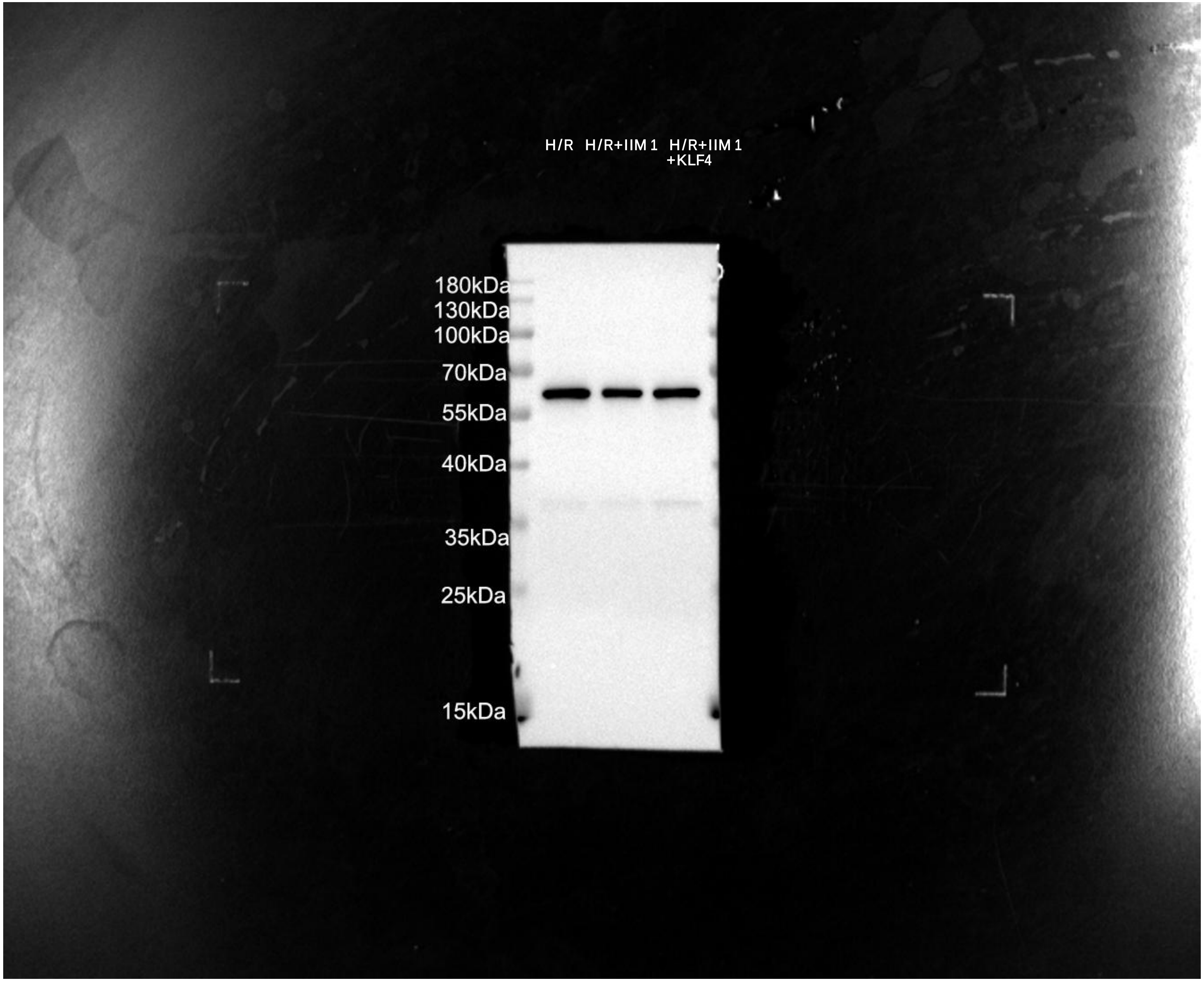

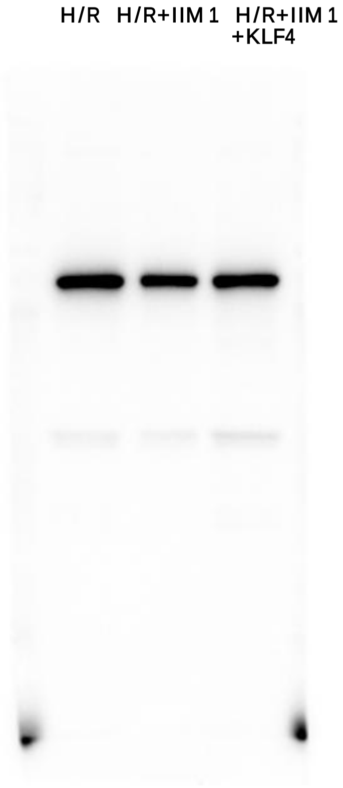
H/R
H/R+IIM 1
H/R+IIM 1
+KLF4

Fig 6D LC3

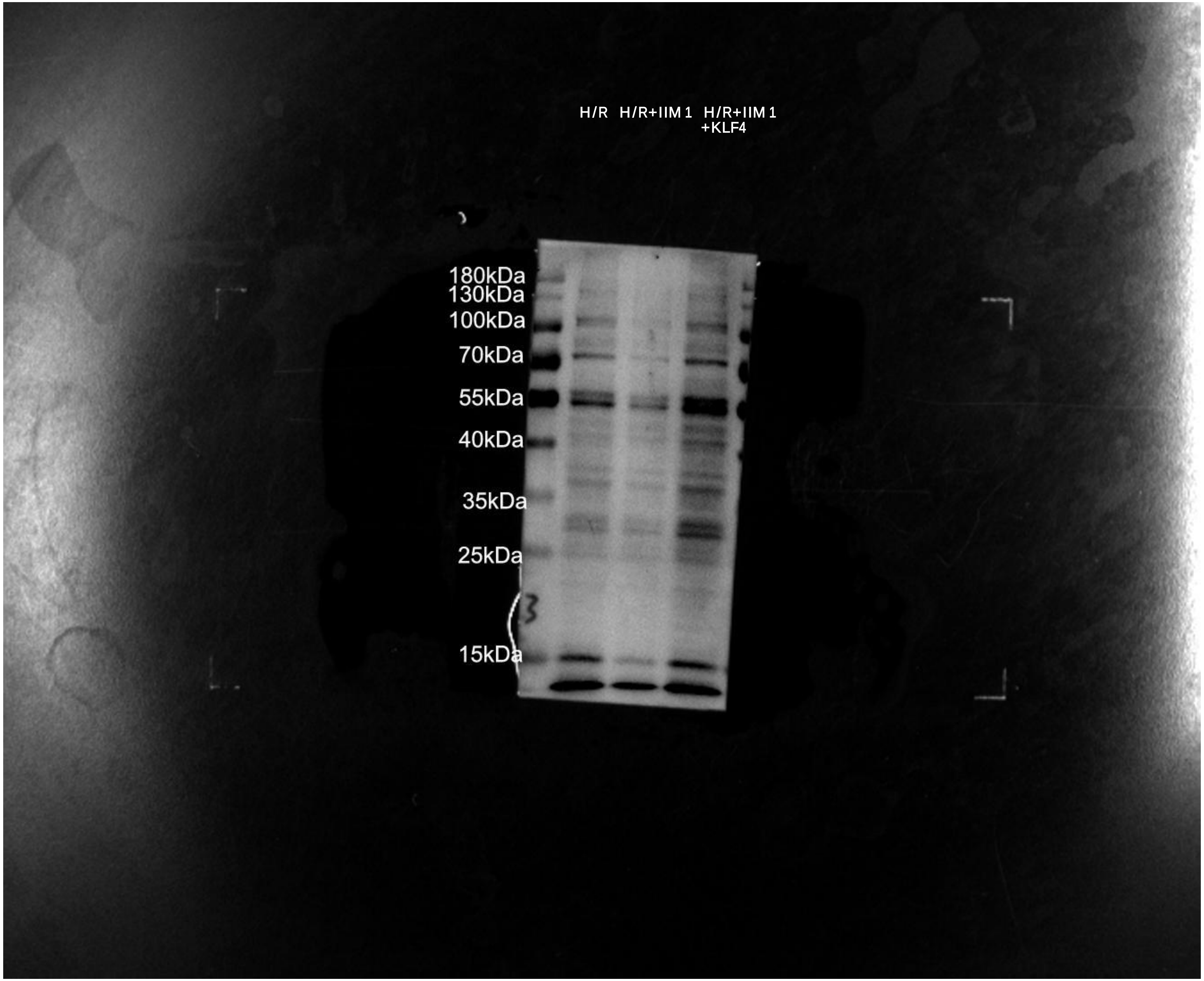
H/R
H/R+IIM1
H/R+IIM1 +KLF4
180kDa
130kDa
100kDa
70kDa
55kDa
40kDa
35kDa
25kDa
15kDa

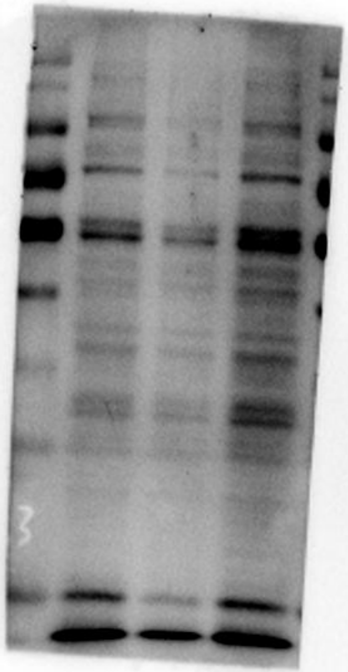
H/R
H/R+IIM 1
H/R+IIM 1 +KLF4
